# Role of Agile in Digital Public Health Transformation

**DOI:** 10.3389/fpubh.2022.899874

**Published:** 2022-05-12

**Authors:** Peter Kokol, Helena Blažun Vošner, Marko Kokol, Jernej Završnik

**Affiliations:** ^1^Faculty of Electrical Engineering and Computer Science, University of Maribor, Maribor, Slovenia; ^2^Faculty of Medicine, University of Maribor, Maribor, Slovenia; ^3^Community Healthcare Center Dr. Adolf Drolc Maribor, Maribor, Slovenia; ^4^Alma Mater Europaea, Maribor, Slovenia; ^5^Faculty of Health and Social Sciences Slovenj Gradec, Slovenj Gradec, Slovenia; ^6^Semantika Research, Semantika d.o.o., Maribor, Slovenia; ^7^Science and Research Centre, Koper, Slovenia; ^8^Faculty of Natural Sciences and Mathematics, University of Maribor, Maribor, Slovenia

**Keywords:** digital health, software development, agile approach, bibliometric, synthetic knowledge synthesis

## Abstract

The digitalisation of healthcare, fueled by advances in technology and the COVID-19 pandemic can not only empower equitable access to global expert-level healthcare but also make healthcare more patient-centric. Every digital health solution has one common fundamental component: they all run on computing platforms and are powered by complex software. Traditional software development life cycles have often failed in designing complex software; consequently, the agile approach was introduced. To assess the role of agile in digital public health transformation, we used the synthetic knowledge synthesis, a triangulation of bibliometric mapping, and thematic analysis to analyse the available literature harvested from PubMed. The analysis showed that the use of the agile approach is underutilised in developing digital health software. Moreover, the study revealed that health organisations did not yet embrace the agile culture and should adapt using innovative agile solutions to deliver clinical value to patients and public health systems. Following the software industry, where agile software development is becoming the mainstream approach also for sensitive and regulated software, it is becoming even more essential that the digital health software development process should be modernised. Furthermore, a shift to agile collaboration, agile decision-making, trial tolerance, active engagement, purposeful technology adoption, knowledge sharing, and an open agile innovation ecosystem must be achieved.

## Introduction: Digital Health Transformation

The digitalization of healthcare, fueled by advances in technology, increased the prevalence of mobile smart devices, smart hospitals (1), and Internet of Medica Things (2), which can not only empower equitable access to global expert-level healthcare (3, 4) but also make health care more patient-centric (5) and value-based (6). During the COVID-19 crisis, the growing demand for telemedicine, mobile health apps, advanced data analytics, and numerous digital health applications have firmly established their role in a modern information society (7) and increased the willingness of health professionals to adopt digital technology into their daily work routines (8). As seen above healthcare is in transitioning from digital innovations to digital transformation, a trend that will only intensify in the future. Hospitals at home, expanded use of artificial intelligence, co-creation of digital health solutions by patients, digital therapeutics, separation of data from the application, and low-code data management are some of the future directions of this digitalisation (9, 10).

## Digital Health Transformation is Driven by Software

Every digital health solution has one common fundamental component; they all run on computing platforms and are powered by software (digital health software; DHSW). As a matter of fact, the Federal Drug Administration (FDA) considers software to be an important part of every medical product, which is integrated widely into digital platforms that serve medical purposes. FDA distinguishes three categories of software namely: software as a medical device (not part of medical hardware), software in a medical device, and software used in the manufacture or maintenance of medical devices (11). Software as a medical device is a relatively new term in healthcare and serves different purposes like diagnosing, modernising care or treatment, disease prevention, mobile well-being application, smartphone digital solution to view medical images and similar (12) or lately artificial intelligence/machine learning-based health care systems (13).

The DHSW must address a variety of multifaced requirements, such as managing multimodal and high dimensional health data, patient safety, cybersecurity, or operating in real-time, in order to help patients survive. Furthermore, the DHSW must adhere to medical regulations, standards, and customisation needs. As a result, it is highly and intrinsically complex and prone to software defects. It is essential that the DHSW is accepted, trusted, and approved by stakeholders, patients, health professionals, other end users, software developers, and regulators to operate effectively, efficiently, and ethically (14).

## Agile is Shaping the Software Development

Traditional software development life cycles (SDLC) have often failed in designing complex software. Thus, a group of developers proposed a new paradigm for software development in 2001 and unveiled it as the Agile Manifesto (15). Manifesto recognises that software development organisations are complex adaptive systems where, whenever during a software development process a decision has to be made, four fundamental values should be taken into account (while there is value in the items on the right, value the item on the left is higher):

- **Individuals and interactions** over processes and tools
- **Working software** over comprehensive documentation
- **Customer collaboration** over contract negotiation
- **Responding to change** by following a plan

## Limited Adoption of ASD in the Digital Health Transformation

Although healthcare organisations can be considered as complex adaptive systems (16) and agile software development (ASD) is increasingly becoming used in developing safety-critical or regulated software (17), there is limited evidence about the use of ASD in DHSW development. Using a synthetic knowledge synthesis approach (18), we reviewed all 248 publications concerning ASD use in DHSW development.

Synthetic knowledge synthesis is a novel synthesis approach based on the triangulation of bibliometric mapping and content analysis. Unlike traditional knowledge synthesis methods, which are usually performed manually, are labour intensive, and are usually limited to a relatively small number of publications, synthetic knowledge synthesis enables one to process several hundreds of publications semi-automatically. The broadness above makes it a natural fit for mapping new research areas of interest or areas that may have received insufficient attention in previous research. Moreover, it enables one to gain new or more holistic insights into such research areas and form perspectives and solutions to not yet solved barriers and challenges. The following steps are taken when using synthetic knowledge synthesis with the PubMed bibliographic database:

1) Harvest the research publications from the PubMed bibliographic database using the selected search string. It was selected as a source bibliographic database because it contains a wide range of strictly selected health care source titles, is free to use, and allows users to export publication metadata in larger packages (10,000 publications) than other platforms (such as Web of Science, which only allows exporting 500 publications at a time).
2) Select Mesh terms (Medical Subject Headings) as meaningful information units. Mesh terms were selected because they are standardised keywords representing the content of the publication.
3) Map Mesh terms using bibliometric mapping into a cluster landscape.
4) Analyse the links and proximity between Mesh terms in individual clusters and form categories.
5) Condense categories into themes.

We used VOSViewer software (Leiden University, Netherlands) as a bibliometric mapping tool, and search string agile AND (software OR “information system” OR digital) AND (health^*^ OR medic^*^ *OR nursing)*. The search was performed on the 15th of February. Papers without abstracts and not related to humans or software development were discarded.

**Figure 1:**
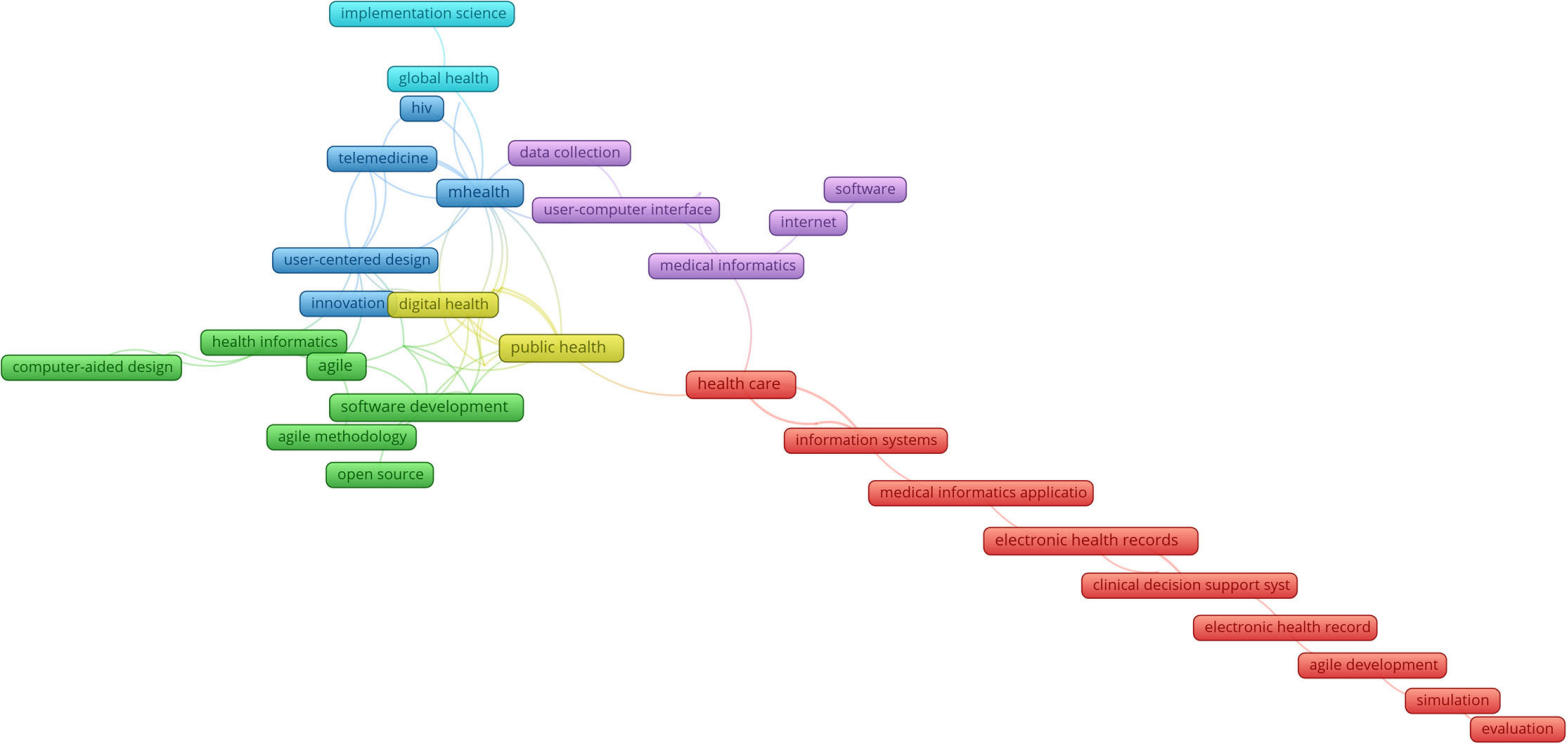
Agile software development (ASD) uses digital health software (DHSW) development.

The synthesis revealed that research on the ASD use in health care revolves around six themes (Figure 1): development of open source and clinical decision support software (green colour); e-health applications for COVID-19 management (yellow colour); user-centred design of mHealth and telemedicine application for chronic diseases management (blue colour); solving global health problems (light blue colour); user—computer interfaces for health care data-collection (violet colour); and information management of patients data and decision making (red colour). The adaption focuses on a few from a broad scope of digital health areas, namely health information systems, decision support, and mobile applications. Most other areas still favour the right side of the Agile manifesto values and rely on traditional SDLCs. Moreover, the volume of research is not sufficient, especially when taking into account the ever-increasing importance of the DHSW role in improving the performance of health care systems.

In also taking into account the grey literature, we identified some barriers to limited use of ASD in the DHSW development:

- Health Insurance Portability and Accountability Act compliance when developing embedded DHSW;
- Securing Protected Health Information when writing User stories (User stories replace Traditional requirements specifications in ASD);
- Managing a high volume of stakeholders, which is a unique characteristic in developing DHSW and not suitable for ASD;
- Interoperability DHSW, where the requirements are well-defined and non-negotiable, and thus more appropriate for the use of traditional SDLCs

## Agile Beyond DHSW Development

As digital health and digital health technologies gain popularity and healthcare costs continue to rise, healthcare organisations are being forced to find innovative solutions that deliver new patient and clinician value. Hence, health organisations have no alternative but to adapt. However, their current business planning and governance processes, as well as information and communication technology solutions, are premediated to minimise risk to health organisations, which consequently makes them inflexible and unable to adapt and perform complex, but essential transformations. To manage this complexity and enable transformation, some health care organisations followed the software industry and embraced the principles and values of the Agile Manifesto (19).

As in the DHSW, we used a synthetic knowledge synthesis approach (18) to analyse 210 publications about agile management in digital health found in the PubMed bibliographic database to determine the volume and content of research in this area. We used the search string *agile AND management AND (health OR medicine)*. The search was performed on the 15th of February. Papers without abstracts and not related to humans or management of health organisations were discarded.

**Figure 2:**
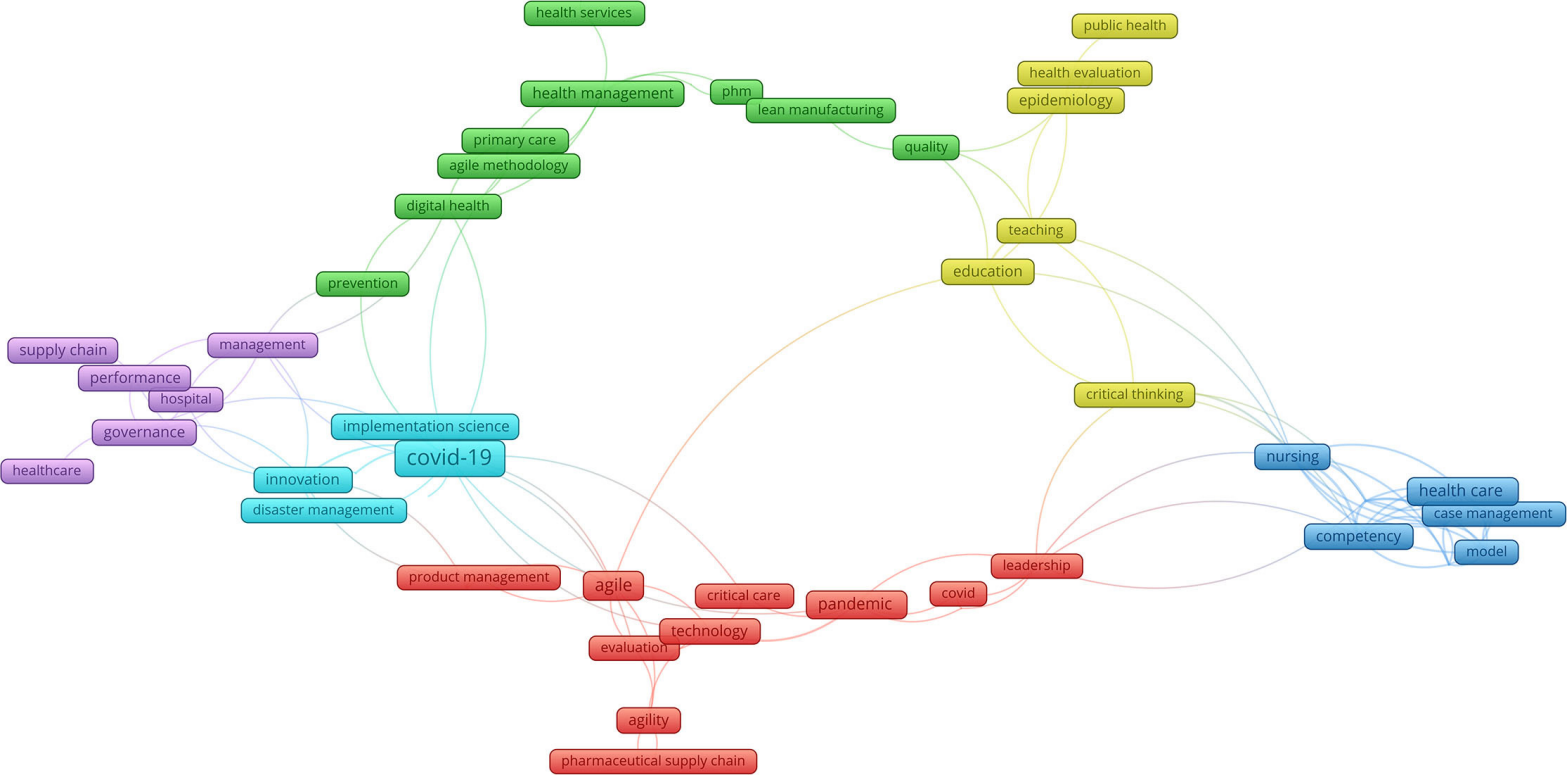
Agile in digital health.

Six colours in the author keywords landscape (Figure 2) represent six agile management themes: agile management of health services in digital health (green colour); critical evaluation of public health and health education (yellow colour); agile trans-disciplinarity in health and social care (blue colour); agile leadership and management in pharmaceutical supply chains (red colour); agile management of innovations in case of COVID-19 (light blue colour); management and governance in healthcare supply chains (violet colour). As in agile in DHSW development agile in management also seems to be underutilised. Adapting health care organisations to embrace agile culture showed to be a challenging task, due to Ahire et al. (20) and Sindhwani et al. (21):

- Lack of patient and employee input and empowerment
- Lack of organisational structure and top management support
- Insufficient communication, cohesion, cooperation, and opposing incentives between stakeholders
- Lack of training

## Discussion: How to Overcome Barriers

While current trends in the software industry, where ASD is becoming the mainstream approach also for sensitive and regulated software, it is becoming even more essential that the DHSW development process should be modernised. Agile, with its proven track record in a broad number of verticals and 20 years after being formalised in the Agile Manifesto, is thus—in our opinion—underutilised and under-researched. ASD combined with modern development approaches, such as Microservices, can be successfully adapted (22) and might efficaciously address the above barriers:

- The rapid development of Proofs of Concepts and Minimal Viable Products is crucial to demonstrate a product to possible stakeholders, incorporate changes in regulatory requirements or react quickly to new technologies and new requirements.
- Enhance testing and quality assurance process through better work breakdown and modularity.
- Provide APIs to other stakeholders quickly to integrate the newly developing software while still in development.
- Use machine learning to better understand critical health care systems' user requirements and stories (23).

Viewing agility as the means to respond quickly to the customer and the environment, and to be faster and more patient-centric is simply not enough (24). According to Gallup (25), health organisations' purposeful agility has to include three key elements, namely adaptability, speed, and execution. To reach the agile culture they must shift to *agile collaboration, agile decision-making, trial tolerance, active engagement, purposeful technology adoption, and knowledge sharing*. Valentim et al. (26) vent even further, namely to enhance the agility of digital health solutions during the COVID-19 pandemics, they developed an innovative technology ecosystem integrating various information systems to enhance governmental decision making. Similarly, to improve the flexibility and adaptability of health organisations during the COVID-19 crisis, due to the lack of knowledge of new viruses, fear of people and ever-changing COVID-19 management strategies, Brunet et al. (27) constructed an agile-based open ecosystem in the manner to progress responds to the crisis. Similar ecosystems were also developed by Samad et al. (28) and interestingly applied for tackling health problems in post-pandemic society by Marston et al. (29).

## Data Availability Statement

The original contributions presented in the study are included in the article/Supplementary Material, further inquiries can be directed to the corresponding author/s.

## Author Contributions

PK prepared the concept and performed the analysis. MK, HB, and JZ interpreted the results and supervised the study. All authors cooperated in writing and reviewing the article.

## Funding

The study was partially funded by the EC H2020 STAMINA project, GA 883441.

## Conflict of Interest

MK was employed by the company Semantika. The remaining authors declare that the research was conducted in the absence of any commercial or financial relationships that could be construed as a potential conflict of interest.

## Publisher's Note

All claims expressed in this article are solely those of the authors and do not necessarily represent those of their affiliated organizations, or those of the publisher, the editors and the reviewers. Any product that may be evaluated in this article, or claim that may be made by its manufacturer, is not guaranteed or endorsed by the publisher.
