# Supplementary material for: Role of Agile in Digital Public Health Transformation
Source: Front Public Health. 2022 May 12;10:899874. doi: 10.3389/fpubh.2022.899874 (PMC9134062; doi:10.3389/fpubh.2022.899874)
Supplement: Supplementary file 1 [file Data_Sheet_1.DOCX]

1: McCarthy GW, Anton DJ, Bayley NJ, Martin TE, Stallard N. The effect of a new

ejection seat headbox and high G garments on head mobility during air combat.

Aviat Space Environ Med. 1994 Mar;65(3):187-92. PMID: 8185545.

2: Carrico R, Goss L. Syndromic surveillance: hospital emergency department

participation during the Kentucky Derby Festival. Disaster Manag Response. 2005

Jul-Sep;3(3):73-9. doi: 10.1016/j.dmr.2005.04.003. PMID: 15986027; PMCID:

PMC7110958.

3: Yang ZS, Mao BL. [Development of a mobile digital hydraulic extracorporeal

heart compression machine]. Zhongguo Yi Liao Qi Xie Za Zhi. 2005

Jul;29(4):257-9. Chinese. PMID: 16268351.

4: Kitzmiller R, Hunt E, Sproat SB. Adopting best practices: "Agility" moves

from software development to healthcare project management. Comput Inform Nurs.

2006 Mar-Apr;24(2):75-82; quiz 83-4. doi: 10.1097/00024665-200603000-00005.

PMID: 16554690.

5: Kane DW, Hohman MM, Cerami EG, McCormick MW, Kuhlmman KF, Byrd JA. Agile

methods in biomedical software development: a multi-site experience report. BMC

Bioinformatics. 2006 May 30;7:273. doi: 10.1186/1471-2105-7-273. PMID: 16734914;

PMCID: PMC1539031.

6: Burgess L, Sargent J. Enhancing user acceptance of mandated mobile health

information systems: the ePOC (electronic point-of-care project) experience.

Stud Health Technol Inform. 2007;129(Pt 2):1088-92. PMID: 17911883.

7: Kamino Y, Tsukuda K, Kokubo M, Miura S, Hirai E, Hiraoka M, Ishikawa J.

Development of a new concept automatic frequency controller for an ultrasmall

C-band linear accelerator guide. Med Phys. 2007 Aug;34(8):3243-8. doi:

10.1118/1.2752581. PMID: 17879787.

8: Chung J, Pankey E, Norris RJ. Agile informatics: application of agile project

management to the development of a personal health application. AMIA Annu Symp

Proc. 2007 Oct 11:914. PMID: 18694014.

9: Narasimhadevara A, Radhakrishnan T, Leung B, Jayakumar R. On designing a

usable interactive system to support transplant nursing. J Biomed Inform. 2008

Feb;41(1):137-51. doi: 10.1016/j.jbi.2007.03.006. Epub 2007 Mar 31. PMID:

17681889.

10: Barreto FR, Teixeira MG, Costa Mda C, Carvalho MS, Barreto ML. Spread

pattern of the first dengue epidemic in the city of Salvador, Brazil. BMC Public

Health. 2008 Feb 7;8:51. doi: 10.1186/1471-2458-8-51. PMID: 18257919; PMCID:

PMC2287177.

11: Pitt-Francis J, Bernabeu MO, Cooper J, Garny A, Momtahan L, Osborne J,

Pathmanathan P, Rodriguez B, Whiteley JP, Gavaghan DJ. Chaste: using agile

programming techniques to develop computational biology software. Philos Trans A

Math Phys Eng Sci. 2008 Sep 13;366(1878):3111-36. doi: 10.1098/rsta.2008.0096.

PMID: 18565813.

12: Moen A, Smørdal O, Sem I. Web-based resources for peer support -

opportunities and challenges. Stud Health Technol Inform. 2009;150:302-6. PMID:

19745318.

13: Lehne B, Schlitt T. Protein-protein interaction databases: keeping up with

growing interactomes. Hum Genomics. 2009 Apr;3(3):291-7. doi:

10.1186/1479-7364-3-3-291. PMID: 19403463; PMCID: PMC3500230.

14: Vellay SG, Latimer NE, Paillard G. Interactive text mining with Pipeline

Pilot: a bibliographic web-based tool for PubMed. Infect Disord Drug Targets.

2009 Jun;9(3):366-74. doi: 10.2174/1871526510909030366. PMID: 19519489.

15: Krause P, de Lusignan S. Procuring interoperability at the expense of

usability: a case study of UK National Programme for IT assurance process. Stud

Health Technol Inform. 2010;155:143-9. PMID: 20543322.

16: Smith E, Lai JS, Cella D. Building a measure of fatigue: the functional

assessment of Chronic Illness Therapy Fatigue Scale. PM R. 2010 May;2(5):359-63.

doi: 10.1016/j.pmrj.2010.04.017. PMID: 20656617.

17: Zhao J, Yang H, Guo H, Li Y, Zhang Z, Li S. [Research on multi-agent based

modeling and simulation of hospital system]. Sheng Wu Yi Xue Gong Cheng Xue Za

Zhi. 2010 Dec;27(6):1346-9. Chinese. PMID: 21374992.

18: Boussadi A, Bousquet C, Sabatier B, Caruba T, Durieux P, Degoulet P. A

business rules design framework for a pharmaceutical validation and alert

system. Methods Inf Med. 2011;50(1):36-50. doi: 10.3414/ME09-01-0074. Epub 2010

Oct 20. PMID: 20963255.

19: Williamson HM. Disaster management mobile protocols: a technology that will

save lives. Am J Disaster Med. 2011 Jan-Feb;6(1):55-64. PMID: 21466030.

20: Papakonstantinou D, Poulymenopoulou M, Malamateniou F, Vassilacopoulos G. A

cloud-based semantic wiki for user training in healthcare process management.

Stud Health Technol Inform. 2011;169:93-7. PMID: 21893721.

21: Senathirajah Y, Bakken S. Important ingredients for health adaptive

information systems. Stud Health Technol Inform. 2011;169:280-4. PMID: 21893757.

22: Misra S, Agrawal A, Liao WK, Choudhary A. Anatomy of a hash-based long read

sequence mapping algorithm for next generation DNA sequencing. Bioinformatics.

2011 Jan 15;27(2):189-95. doi: 10.1093/bioinformatics/btq648. Epub 2010 Nov 18.

PMID: 21088030.

23: Lopes P, Dalgleish R, Oliveira JL. WAVe: web analysis of the variome. Hum

Mutat. 2011 Jul;32(7):729-34. doi: 10.1002/humu.21499. Epub 2011 Apr 7. PMID:

21394829.

24: Sadasivam RS, Delaughter K, Crenshaw K, Sobko HJ, Williams JH, Coley HL, Ray

MN, Ford DE, Allison JJ, Houston TK. Development of an interactive, Web-

delivered system to increase provider-patient engagement in smoking cessation. J

Med Internet Res. 2011 Oct 18;13(4):e87. doi: 10.2196/jmir.1721. PMID: 22011394;

PMCID: PMC3222193.

25: Tran VA, Johnson N, Redline S, Zhang GQ. OnWARD: ontology-driven web-based

framework for multi-center clinical studies. J Biomed Inform. 2011 Dec;44 Suppl

1(Suppl 1):S48-S53. doi: 10.1016/j.jbi.2011.08.019. Epub 2011 Sep 8. PMID:

21924379; PMCID: PMC3251701.

26: Sahoo SS, Zhao M, Luo L, Bozorgi A, Gupta D, Lhatoo SD, Zhang GQ. OPIC:

Ontology-driven Patient Information Capturing system for epilepsy. AMIA Annu

Symp Proc. 2012;2012:799-808. Epub 2012 Nov 3. PMID: 23304354; PMCID:

PMC3540561.

27: Ramly E, Brennan PF. Guiding the design of evaluations of innovations in

health informatics: a framework and a case study of the SMArt SHARP evaluation.

AMIA Annu Symp Proc. 2012;2012:1375-84. Epub 2012 Nov 3. PMID: 23304417; PMCID:

PMC3540442.

28: Sinard JH, Gershkovich P. Custom software development for use in a clinical

laboratory. J Pathol Inform. 2012;3:44. doi: 10.4103/2153-3539.104906. Epub 2012

Dec 20. PMID: 23372985; PMCID: PMC3551490.

29: Teodoro D, Pasche E, Gobeill J, Emonet S, Ruch P, Lovis C. Building a

transnational biosurveillance network using semantic web technologies:

requirements, design, and preliminary evaluation. J Med Internet Res. 2012 May

29;14(3):e73. doi: 10.2196/jmir.2043. PMID: 22642960; PMCID: PMC3799609.

30: Holmes M. Everything a medical innovator needs to know about developing

software. Australas Med J. 2013;6(1):19-22. doi: 10.4066/AMJ.2013.1579. Epub

2013 Jan 31. PMID: 23424045; PMCID: PMC3575062.

31: Choi J. Exploring the possibility of modeling a genetic counseling guideline

using agile methodology. Stud Health Technol Inform. 2013;192:1200. PMID:

23920974.

32: Gonzales MJ, O'Connor MF, Riek LD. Contextual constraints for the design of

patient-centered health IT tools. Stud Health Technol Inform. 2013;194:75-81.

PMID: 23941934.

33: Ellis PG. Development and implementation of oncology care pathways in an

integrated care network: the Via Oncology Pathways experience. J Oncol Pract.

2013 May;9(3):171-3. doi: 10.1200/JOP.2013.001020. PMID: 23942503; PMCID:

PMC3651572.

34: Carr IM, Morgan J, Watson C, Melnik S, Diggle CP, Logan CV, Harrison SM,

Taylor GR, Pena SD, Markham AF, Alkuraya FS, Black GC, Ali M, Bonthron DT.

Simple and efficient identification of rare recessive pathologically important

sequence variants from next generation exome sequence data. Hum Mutat. 2013

Jul;34(7):945-52. doi: 10.1002/humu.22322. Epub 2013 Apr 29. PMID: 23554237.

35: Savel TG, Lee BA, Ledbetter G, Brown S, Lavalley D, Taylor J, Thompson P.

PTT Advisor: A CDC-supported initiative to develop a mobile clinical laboratory

decision support application for the iOS platform. Online J Public Health

Inform. 2013 Jul 1;5(2):215. doi: 10.5210/ojphi.v5i2.4363. PMID: 23923100;

PMCID: PMC3733759.

36: Spreckelsen C, Finsterer S, Cremer J, Schenkat H. Can social semantic web

techniques foster collaborative curriculum mapping in medicine? J Med Internet

Res. 2013 Aug 15;15(8):e169. doi: 10.2196/jmir.2623. PMID: 23948519; PMCID:

PMC3758046.

37: Gaspar P, Lopes P, Oliveira J, Santos R, Dalgleish R, Oliveira JL. Variobox:

automatic detection and annotation of human genetic variants. Hum Mutat. 2014

Feb;35(2):202-7. doi: 10.1002/humu.22474. Epub 2013 Nov 21. PMID: 24186831.

38: Duke JD, Morea J, Mamlin B, Martin DK, Simonaitis L, Takesue BY, Dixon BE,

Dexter PR. Regenstrief Institute's Medical Gopher: a next-generation homegrown

electronic medical record system. Int J Med Inform. 2014 Mar;83(3):170-9. doi:

10.1016/j.ijmedinf.2013.11.004. Epub 2013 Dec 14. PMID: 24373714.

39: Schutzbank A, Fernandopulle R. Doubling down: Lessons learned from building

a new electronic health record as part of primary care practice redesign.

Healthc (Amst). 2014 Mar;2(1):14-8. doi: 10.1016/j.hjdsi.2013.12.007. Epub 2014

Mar 18. PMID: 26250083.

40: Smits R, Bryant J, Sanson-Fisher R, Tzelepis F, Henskens F, Paul C,

Stevenson W. Tailored and integrated Web-based tools for improving psychosocial

outcomes of cancer patients: the DoTTI development framework. J Med Internet

Res. 2014 Mar 14;16(3):e76. doi: 10.2196/jmir.2849. PMID: 24641991; PMCID:

PMC3971205.

41: van Mierlo T, Fournier R, Jean-Charles A, Hovington J, Ethier I, Selby P.

I'll txt U if I have a problem: how the Société Canadienne du cancer in Quebec

applied behavior-change theory, data mining and agile software development to

help young adults quit smoking. PLoS One. 2014 Mar 19;9(3):e91832. doi:

10.1371/journal.pone.0091832. PMID: 24647098; PMCID: PMC3960136.

42: Birkinshaw J. Beware the next big thing. Harv Bus Rev. 2014 May;92(5):50-7,

134. PMID: 24956869.

43: Macfarlane MA. Sustainable competitive advantage for accountable care

organizations. J Healthc Manag. 2014 Jul-Aug;59(4):263-71. doi:

10.1097/00115514-201407000-00006. PMID: 25154124.

44: Bridge P, Gunn T, Kastanis L, Pack D, Rowntree P, Starkey D, Mahoney G,

Berry C, Braithwaite V, Wilson-Stewart K. The development and evaluation of a

medical imaging training immersive environment. J Med Radiat Sci. 2014

Sep;61(3):159-65. doi: 10.1002/jmrs.60. Epub 2014 Jun 30. Erratum in: J Med

Radiat Sci. 2014 Dec;61(4):279. PMID: 26229652; PMCID: PMC4175849.

45: Horvath MM, Rusincovitch SA, Brinson S, Shang HC, Evans S, Ferranti JM.

Modular design, application architecture, and usage of a self-service model for

enterprise data delivery: the Duke Enterprise Data Unified Content Explorer

(DEDUCE). J Biomed Inform. 2014 Dec;52:231-42. doi: 10.1016/j.jbi.2014.07.006.

Epub 2014 Jul 19. PMID: 25051403; PMCID: PMC4335712.

46: Kawamoto K, Martin CJ, Williams K, Tu MC, Park CG, Hunter C, Staes CJ, Bray

BE, Deshmukh VG, Holbrook RA, Morris SJ, Fedderson MB, Sletta A, Turnbull J,

Mulvihill SJ, Crabtree GL, Entwistle DE, McKenna QL, Strong MB, Pendleton RC,

Lee VS. Value Driven Outcomes (VDO): a pragmatic, modular, and extensible

software framework for understanding and improving health care costs and

outcomes. J Am Med Inform Assoc. 2015 Jan;22(1):223-35. doi:

10.1136/amiajnl-2013-002511. Epub 2014 Oct 16. PMID: 25324556; PMCID:

PMC4433359.

47: Kushniruk AW, Borycki EM. Integrating Low-Cost Rapid Usability Testing into

Agile System Development of Healthcare IT: A Methodological Perspective. Stud

Health Technol Inform. 2015;210:200-4. PMID: 25991130.

48: Koufi V, Malamateniou F, Vassilacopoulos G. A Big Data-driven Model for the

Optimization of Healthcare Processes. Stud Health Technol Inform.

2015;210:697-701. PMID: 25991242.

49: Banos O, Villalonga C, Garcia R, Saez A, Damas M, Holgado-Terriza JA, Lee S,

Pomares H, Rojas I. Design, implementation and validation of a novel open

framework for agile development of mobile health applications. Biomed Eng

Online. 2015;14 Suppl 2(Suppl 2):S6. doi: 10.1186/1475-925X-14-S2-S6. Epub 2015

Aug 13. PMID: 26329639; PMCID: PMC4547155.

50: Raghu A, Praveen D, Peiris D, Tarassenko L, Clifford G. Engineering a mobile

health tool for resource-poor settings to assess and manage cardiovascular

disease risk: SMARThealth study. BMC Med Inform Decis Mak. 2015 Apr 29;15:36.

doi: 10.1186/s12911-015-0148-4. PMID: 25924825; PMCID: PMC4430914.

51: Årsand E, Muzny M, Bradway M, Muzik J, Hartvigsen G. Performance of the

first combined smartwatch and smartphone diabetes diary application study. J

Diabetes Sci Technol. 2015 May;9(3):556-63. doi: 10.1177/1932296814567708. Epub

2015 Jan 14. PMID: 25591859; PMCID: PMC4604524.

52: Melnick ER, Lopez K, Hess EP, Abujarad F, Brandt CA, Shiffman RN, Post LA.

Back to the Bedside: Developing a Bedside Aid for Concussion and Brain Injury

Decisions in the Emergency Department. EGEMS (Wash DC). 2015 Jun 29;3(2):1136.

doi: 10.13063/2327-9214.1136. PMID: 26290885; PMCID: PMC4537154.

53: Morrison D, Mair FS, Chaudhuri R, McGee-Lennon M, Thomas M, Thomson NC,

Yardley L, Wyke S. Details of development of the resource for adults with asthma

in the RAISIN (randomized trial of an asthma internet self-management

intervention) study. BMC Med Inform Decis Mak. 2015 Jul 28;15:57. doi:

10.1186/s12911-015-0177-z. PMID: 26215651; PMCID: PMC4517557.

54: Buckingham CD, Adams A, Vail L, Kumar A, Ahmed A, Whelan A, Karasouli E.

Integrating service user and practitioner expertise within a web-based system

for collaborative mental-health risk and safety management. Patient Educ Couns.

2015 Oct;98(10):1189-96. doi: 10.1016/j.pec.2015.08.018. Epub 2015 Aug 14. PMID:

26320825.

55: Wright A, Sittig DF, Ash JS, Erickson JL, Hickman TT, Paterno M, Gebhardt E,

McMullen C, Tsurikova R, Dixon BE, Fraser G, Simonaitis L, Sonnenberg FA,

Middleton B. Lessons learned from implementing service-oriented clinical

decision support at four sites: A qualitative study. Int J Med Inform. 2015

Nov;84(11):901-11. doi: 10.1016/j.ijmedinf.2015.08.008. Epub 2015 Aug 20. PMID:

26343972.

56: Cordova D, Bauermeister JA, Fessler K, Delva J, Nelson A, Nurenberg R,

Mendoza Lua F, Alers-Rojas F, Salas-Wright CP; Youth Leadership Council. A

Community-Engaged Approach to Developing an mHealth HIV/STI and Drug Abuse

Preventive Intervention for Primary Care: A Qualitative Study. JMIR Mhealth

Uhealth. 2015 Dec 18;3(4):e106. doi: 10.2196/mhealth.4620. PMID: 26685288;

PMCID: PMC4704933.

57: Lindoerfer D, Mansmann U. Proposing an Evidence-Based Strategy for Software

Requirements Engineering. Stud Health Technol Inform. 2016;228:648-52. PMID:

27577464.

58: Mellor N, Horton H, Luke D, Meadows J, Chatterjee A, Gale T. Experience of

Using Simulation Technology and Analytics During the Ebola Crisis to Empower

Frontline Health Workers and Improve the Integrity of Public Health Systems.

Procedia Eng. 2016;159:44-52. doi: 10.1016/j.proeng.2016.08.062. Epub 2016 Sep

14. PMID: 32288922; PMCID: PMC7130016.

59: Silva GG, Green KT, Dutilh BE, Edwards RA. SUPER-FOCUS: a tool for agile

functional analysis of shotgun metagenomic data. Bioinformatics. 2016 Feb

1;32(3):354-61. doi: 10.1093/bioinformatics/btv584. Epub 2015 Oct 9. PMID:

26454280; PMCID: PMC4734042.

60: McDowell J, Styles K, Sewell K, Trinder P, Marriott J, Maher S, Naidu S. A

Simulated Learning Environment for Teaching Medicine Dispensing Skills. Am J

Pharm Educ. 2016 Feb 25;80(1):11. doi: 10.5688/ajpe80111. PMID: 26941437; PMCID:

PMC4776289.

61: Neinstein A, Wong J, Look H, Arbiter B, Quirk K, McCanne S, Sun Y, Blum M,

Adi S. A case study in open source innovation: developing the Tidepool Platform

for interoperability in type 1 diabetes management. J Am Med Inform Assoc. 2016

Mar;23(2):324-32. doi: 10.1093/jamia/ocv104. Epub 2015 Sep 2. PMID: 26338218;

PMCID: PMC4784555.

62: Militello LG, Saleem JJ, Borders MR, Sushereba CE, Haverkamp D, Wolf SP,

Doebbeling BN. Designing Colorectal Cancer Screening Decision Support: A

Cognitive Engineering Enterprise. J Cogn Eng Decis Mak. 2016 Mar;10(1):74-90.

doi: 10.1177/1555343416630875. PMID: 26973441; PMCID: PMC4784691.

63: Tweya H, Feldacker C, Gadabu OJ, Ng'ambi W, Mumba SL, Phiri D, Kamvazina L,

Mwakilama S, Kanyerere H, Keiser O, Mwafilaso J, Kamba C, Egger M, Jahn A,

Simwaka B, Phiri S. Developing a point-of-care electronic medical record system

for TB/HIV co-infected patients: experiences from Lighthouse Trust, Lilongwe,

Malawi. BMC Res Notes. 2016 Mar 5;9:146. doi: 10.1186/s13104-016-1943-4. PMID:

26945749; PMCID: PMC4779573.

64: Gale TC, Chatterjee A, Mellor NE, Allan RJ. Health Worker Focused

Distributed Simulation for Improving Capability of Health Systems in Liberia.

Simul Healthc. 2016 Apr;11(2):75-81. doi: 10.1097/SIH.0000000000000156. PMID:

27043091.

65: Flood D, Chary A, Austad K, Diaz AK, García P, Martinez B, Canú WL, Rohloff

P. Insights into Global Health Practice from the Agile Software Development

Movement. Glob Health Action. 2016 Apr 29;9:29836. doi: 10.3402/gha.v9.29836.

PMID: 27134081; PMCID: PMC4852203.

66: Greenberg AE, Hays H, Castel AD, Subramanian T, Happ LP, Jaurretche M,

Binkley J, Kalmin MM, Wood K, Hart R; DC Cohort Executive Committee. Development

of a large urban longitudinal HIV clinical cohort using a web-based platform to

merge electronically and manually abstracted data from disparate medical record

systems: technical challenges and innovative solutions. J Am Med Inform Assoc.

2016 May;23(3):635-43. doi: 10.1093/jamia/ocv176. Epub 2015 Dec 31. PMID:

26721732; PMCID: PMC4901378.

67: Shats O, Goldner W, Feng J, Sherman A, Smith RB, Sherman S. Thyroid Cancer

and Tumor Collaborative Registry (TCCR). Cancer Inform. 2016 May 3;15:73-9. doi:

10.4137/CIN.S32470. PMID: 27168721; PMCID: PMC4856228.

68: Karssen LC, van Duijn CM, Aulchenko YS. The GenABEL Project for statistical

genomics. F1000Res. 2016 May 19;5:914. doi: 10.12688/f1000research.8733.1. PMID:

27347381; PMCID: PMC4916982.

69: Hekler EB, Klasnja P, Riley WT, Buman MP, Huberty J, Rivera DE, Martin CA.

Agile science: creating useful products for behavior change in the real world.

Transl Behav Med. 2016 Jun;6(2):317-28. doi: 10.1007/s13142-016-0395-7. PMID:

27357001; PMCID: PMC4927453.

70: Modave F, Bian J, Rosenberg E, Mendoza T, Liang Z, Bhosale R, Maeztu C,

Rodriguez C, Cardel MI. DiaFit: The Development of a Smart App for Patients with

Type 2 Diabetes and Obesity. JMIR Diabetes. 2016 Jul-Dec;1(2):e5. doi:

10.2196/diabetes.6662. PMID: 29388609; PMCID: PMC5788459.

71: Mishuris RG, Yoder J, Wilson D, Mann D. Integrating data from an online

diabetes prevention program into an electronic health record and clinical

workflow, a design phase usability study. BMC Med Inform Decis Mak. 2016 Jul

11;16:88. doi: 10.1186/s12911-016-0328-x. PMID: 27401606; PMCID: PMC4940704.

72: Ahern DK, Parker D, Eaton C, Rafferty C, Wroblewski J, Goldman R. Patient-

facing Technology for Identification of COPD in Primary Care. J Innov Health

Inform. 2016 Jul 15;23(2):824. doi: 10.14236/jhi.v23i2.824. PMID: 27869583.

73: Lennerz JK, McLaughlin HM, Baron JM, Rasmussen D, Sumbada Shin M, Berners-

Lee N, Miller Batten J, Swoboda KJ, Gala MK, Winter HS, Schmahmann JD, Sweetser

DA, Boswell M, Pacula M, Stenzinger A, Le LP, Hynes W, Rehm HL, Klibanski A,

Black-Schaffer SW, Golden JA, Louis DN, Weiss ST, Iafrate AJ. Health Care

Infrastructure for Financially Sustainable Clinical Genomics. J Mol Diagn. 2016

Sep;18(5):697-706. doi: 10.1016/j.jmoldx.2016.04.003. Epub 2016 Jul 25. PMID:

27471182; PMCID: PMC5397703.

74: Rohatagi S, Profit D, Hatch A, Zhao C, Docherty JP, Peters-Strickland TS.

Optimization of a Digital Medicine System in Psychiatry. J Clin Psychiatry. 2016

Sep;77(9):e1101-e1107. doi: 10.4088/JCP.16m10693. PMID: 27487251.

75: Patrick K, Hekler EB, Estrin D, Mohr DC, Riper H, Crane D, Godino J, Riley

WT. The Pace of Technologic Change: Implications for Digital Health Behavior

Intervention Research. Am J Prev Med. 2016 Nov;51(5):816-824. doi:

10.1016/j.amepre.2016.05.001. PMID: 27745681.

76: Ap Dafydd D, Williamson R, Blunt P, Blunt DM. Development of training-

related health care software by a team of clinical educators: their experience,

from conception to piloting. Adv Med Educ Pract. 2016 Nov 7;7:635-640. doi:

10.2147/AMEP.S108426. PMID: 27853396; PMCID: PMC5106184.

77: Abril-Gonzalez M, Portilla FA, Jaramillo-Mejia MC. Standard Health Level

Seven for Odontological Digital Imaging. Telemed J E Health. 2017

Jan;23(1):63-70. doi: 10.1089/tmj.2015.0251. Epub 2016 Jun 1. PMID: 27248059;

PMCID: PMC5240002.

78: Pitkänen J, Nieminen M. AIM - Agile Instrumented Monitoring for Improving

User Experience of Participation in HealthIT Development. Stud Health Technol

Inform. 2017;234:269-274. PMID: 28186053.

79: Albornoz MA, Márquez S, Rubin L, Luna D. Design of a Mobile Application for

Transfusion Medicine. Stud Health Technol Inform. 2017;245:994-998. PMID:

29295250.

80: Phanareth K, Vingtoft S, Christensen AS, Nielsen JS, Svenstrup J, Berntsen

GK, Newman SP, Kayser L. The Epital Care Model: A New Person-Centered Model of

Technology-Enabled Integrated Care for People With Long Term Conditions. JMIR

Res Protoc. 2017 Jan 16;6(1):e6. doi: 10.2196/resprot.6506. PMID: 28093379;

PMCID: PMC5282450.

81: Ray P, Li J, Ariani A, Kapadia V. Tablet-Based Well-Being Check for the

Elderly: Development and Evaluation of Usability and Acceptability. JMIR Hum

Factors. 2017 May 12;4(2):e12. doi: 10.2196/humanfactors.7240. PMID: 28500017;

PMCID: PMC5446664.

82: Aakre CA, Kitson JE, Li M, Herasevich V. Iterative User Interface Design for

Automated Sequential Organ Failure Assessment Score Calculator in Sepsis

Detection. JMIR Hum Factors. 2017 May 18;4(2):e14. doi:

10.2196/humanfactors.7567. PMID: 28526675; PMCID: PMC5454218.

83: G C C L Cardenas R, D Linhares N, L Ferreira R, Pena SDJ. Mendel,MD: A user-

friendly open-source web tool for analyzing WES and WGS in the diagnosis of

patients with Mendelian disorders. PLoS Comput Biol. 2017 Jun 8;13(6):e1005520.

doi: 10.1371/journal.pcbi.1005520. PMID: 28594829; PMCID: PMC5464533.

84: Kannan V, Fish JS, Mutz JM, Carrington AR, Lai K, Davis LS, Youngblood JE,

Rauschuber MR, Flores KA, Sara EJ, Bhat DG, Willett DL. Rapid Development of

Specialty Population Registries and Quality Measures from Electronic Health

Record Data*. An Agile Framework. Methods Inf Med. 2017 Jun 14;56(99):e74-e83.

doi: 10.3414/ME16-02-0031. PMID: 28930362; PMCID: PMC5608102.

85: Oza S, Jazayeri D, Teich JM, Ball E, Nankubuge PA, Rwebembera J, Wing K,

Sesay AA, Kanter AS, Ramos GD, Walton D, Cummings R, Checchi F, Fraser HS.

Development and Deployment of the OpenMRS-Ebola Electronic Health Record System

for an Ebola Treatment Center in Sierra Leone. J Med Internet Res. 2017 Aug

21;19(8):e294. doi: 10.2196/jmir.7881. PMID: 28827211; PMCID: PMC5583502.

86: Kannan V, Basit MA, Youngblood JE, Bryson TD, Toomay SM, Fish JS, Willett

DL. Agile Co-Development for Clinical Adoption and Adaptation of Innovative

Technologies. Health Innov Point Care Conf. 2017 Nov;2018:56-59. doi:

10.1109/HIC.2017.8227583. PMID: 30364762; PMCID: PMC6197812.

87: Iribarren S, Demiris G, Lober B, Chirico C. What Do Patients and Experts

Want in a Smartphone-Based Application to Support Tuberculosis Treatment

Completion? Stud Health Technol Inform. 2018;250:32. PMID: 29857363.

88: Bai A, Mork H, Stray V. How Agile Teams Regard and Practice Universal Design

During Software Development. Stud Health Technol Inform. 2018;256:171-184. PMID:

30371472.

89: Ortiz-Ruiz A, Postigo M, Gil-Casanova S, Cuadrado D, Bautista JM, Rubio JM,

Luengo-Oroz M, Linares M. Plasmodium species differentiation by non-expert on-

line volunteers for remote malaria field diagnosis. Malar J. 2018 Jan

30;17(1):54. doi: 10.1186/s12936-018-2194-8. PMID: 29378588; PMCID: PMC5789591.

90: Hides L, Quinn C, Cockshaw W, Stoyanov S, Zelenko O, Johnson D,

Tjondronegoro D, Quek LH, Kavanagh DJ. Efficacy and outcomes of a mobile app

targeting alcohol use in young people. Addict Behav. 2018 Feb;77:89-95. doi:

10.1016/j.addbeh.2017.09.020. Epub 2017 Sep 29. PMID: 28992580.

91: Tang T, Lim ME, Mansfield E, McLachlan A, Quan SD. Clinician user

involvement in the real world: Designing an electronic tool to improve

interprofessional communication and collaboration in a hospital setting. Int J

Med Inform. 2018 Feb;110:90-97. doi: 10.1016/j.ijmedinf.2017.11.011. Epub 2017

Nov 22. PMID: 29331258.

92: Dale J, Loew J, Nanton V, Grason Smith G. Coproduction of a Theory-Based

Digital Resource for Unpaid Carers (The Care Companion): Mixed-Methods Study.

JMIR Aging. 2018 Feb 28;1(1):e1. doi: 10.2196/aging.9025. PMID: 31518238; PMCID:

PMC6716079.

93: Lacey Bryant S, Bingham H, Carlyle R, Day A, Ferguson L, Stewart D. Forward

view: advancing health library and knowledge services in England. Health Info

Libr J. 2018 Mar;35(1):70-77. doi: 10.1111/hir.12206. Epub 2018 Jan 10. PMID:

29322613.

94: Basit MA, Baldwin KL, Kannan V, Flahaven EL, Parks CJ, Ott JM, Willett DL.

Agile Acceptance Test-Driven Development of Clinical Decision Support

Advisories: Feasibility of Using Open Source Software. JMIR Med Inform. 2018 Apr

13;6(2):e23. doi: 10.2196/medinform.9679. PMID: 29653922; PMCID: PMC5924365.

95: Whiteley L, Brown L, Lally M, Heck N, van den Berg JJ. A Mobile Gaming

Intervention to Increase Adherence to Antiretroviral Treatment for Youth Living

With HIV: Development Guided by the Information, Motivation, and Behavioral

Skills Model. JMIR Mhealth Uhealth. 2018 Apr 23;6(4):e96. doi:

10.2196/mhealth.8155. PMID: 29685863; PMCID: PMC5993532.

96: Tamblyn R, Winslade N, Lee TC, Motulsky A, Meguerditchian A, Bustillo M,

Elsayed S, Buckeridge DL, Couture I, Qian CJ, Moraga T, Huang A. Improving

patient safety and efficiency of medication reconciliation through the

development and adoption of a computer-assisted tool with automated electronic

integration of population-based community drug data: the RightRx project. J Am

Med Inform Assoc. 2018 May 1;25(5):482-495. doi: 10.1093/jamia/ocx107. PMID:

29040609; PMCID: PMC6018649.

97: Wu DTY, Zheng K, Bradley DJ. CHCi - A Dynamic Data Platform for Clinical

Data Capture and Use. AMIA Jt Summits Transl Sci Proc. 2018 May 18;2017:246-255.

PMID: 29888081; PMCID: PMC5961796.

98: de Ramón-Fernández A, Ruiz-Fernández D, Marcos-Jorquera D, Gilart-Iglesias

V. A Distributed Model for Stressors Monitoring Based on Environmental Smart

Sensors. Sensors (Basel). 2018 Jun 14;18(6):1935. doi: 10.3390/s18061935. PMID:

29903981; PMCID: PMC6022167.

99: Gopalakrishnan V, Jha K, Xun G, Ngo HQ, Zhang A. Towards self-learning based

hypotheses generation in biomedical text domain. Bioinformatics. 2018 Jun

15;34(12):2103-2115. doi: 10.1093/bioinformatics/btx837. PMID: 29293920.

100: Giordanengo A, Øzturk P, Hansen AH, Årsand E, Grøttland A, Hartvigsen G.

Design and Development of a Context-Aware Knowledge-Based Module for Identifying

Relevant Information and Information Gaps in Patients With Type 1 Diabetes Self-

Collected Health Data. JMIR Diabetes. 2018 Jul 11;3(3):e10431. doi:

10.2196/10431. PMID: 30291097; PMCID: PMC6238884.

101: Dietrich D, Bornet Dit Vorgeat H, Mazouri S, Ligier Q, Geissbuhler A.

[Moving towards patient-centered care using a mobile application : the Concerto

project]. Rev Med Suisse. 2018 Sep 5;14(617):1543-1547. French. PMID: 30226669.

102: Fisher AM, Mtonga TM, Espino JU, Jonkman LJ, Connor SE, Cappella NK,

Douglas GP. User-centered design and usability testing of RxMAGIC: a

prescription management and general inventory control system for free clinic

dispensaries. BMC Health Serv Res. 2018 Sep 10;18(1):703. doi:

10.1186/s12913-018-3517-8. PMID: 30200939; PMCID: PMC6131751.

103: Leightley D, Puddephatt JA, Jones N, Mahmoodi T, Chui Z, Field M, Drummond

C, Rona RJ, Fear NT, Goodwin L. A Smartphone App and Personalized Text Messaging

Framework (InDEx) to Monitor and Reduce Alcohol Use in Ex-Serving Personnel:

Development and Feasibility Study. JMIR Mhealth Uhealth. 2018 Sep

11;6(9):e10074. doi: 10.2196/10074. PMID: 30206054; PMCID: PMC6231744.

104: Wilson K, Bell C, Wilson L, Witteman H. Agile research to complement agile

development: a proposal for an mHealth research lifecycle. NPJ Digit Med. 2018

Sep 13;1:46. doi: 10.1038/s41746-018-0053-1. PMID: 31304326; PMCID: PMC6550198.

105: Roberts M. Successful Public Health Information System Database Integration

Projects: A Qualitative Study. Online J Public Health Inform. 2018 Sep

21;10(2):e207. doi: 10.5210/ojphi.v10i2.9221. PMID: 30349625; PMCID: PMC6194103.

106: Backman C, Harley A, Peyton L, Kuziemsky C, Mercer J, Monahan MA, Schmidt

S, Singh H, Gravelle D. Development of a Path to Home Mobile App for the

Geriatric Rehabilitation Program at Bruyère Continuing Care: Protocol for User-

Centered Design and Feasibility Testing Studies. JMIR Res Protoc. 2018 Sep

24;7(9):e11031. doi: 10.2196/11031. PMID: 30249591; PMCID: PMC6231760.

107: Han Q, Yang D. Hierarchical Information Entropy System Model for TWfMS.

Entropy (Basel). 2018 Sep 24;20(10):732. doi: 10.3390/e20100732. PMID: 33265821;

PMCID: PMC7512295.

108: Asgari I. Development an Electronic Oral Health Record application for

educational dental setting. J Educ Health Promot. 2018 Oct 29;7:124. doi:

10.4103/jehp.jehp_177_17. PMID: 30505852; PMCID: PMC6225390.

109: Pillalamarri SS, Huyett LM, Abdel-Malek A. Novel Bluetooth-Enabled Tubeless

Insulin Pump: A User Experience Design Approach for a Connected Digital Diabetes

Management Platform. J Diabetes Sci Technol. 2018 Nov;12(6):1132-1142. doi:

10.1177/1932296818804802. Epub 2018 Oct 11. PMID: 30304951; PMCID: PMC6232743.

110: Goodarzi B, Shakeri K, Ghaniyoun A, Heidari M. Assessment correlation of

the organizational agility of human resources with the performance staff of

Tehran Emergency Center. J Educ Health Promot. 2018 Nov 27;7:142. doi:

10.4103/jehp.jehp_109_18. PMID: 30596114; PMCID: PMC6282496.

111: Mann DM, Chokshi SK, Kushniruk A. Bridging the Gap Between Academic

Research and Pragmatic Needs in Usability: A Hybrid Approach to Usability

Evaluation of Health Care Information Systems. JMIR Hum Factors. 2018 Nov

28;5(4):e10721. doi: 10.2196/10721. PMID: 30487119; PMCID: PMC6291682.

112: Ienca M, Vayena E. Dual use in the 21st century: emerging risks and global

governance. Swiss Med Wkly. 2018 Dec 2;148:w14688. doi: 10.4414/smw.2018.14688.

PMID: 30552855.

113: Besiso A, Patrick JD, Dip G, Ho V, Cheng Y. The Impact of an Enterprise

Electronic Medical Record (EEMR) Model vs a Clinical Information System (CIS)

Model on Usability, Efficiency, and Adaptability. AMIA Annu Symp Proc. 2018 Dec

5;2018:242-251. PMID: 30815062; PMCID: PMC6371262.

114: Turner T, Steele E, Mavergames C, Elliott J; Project Transform Team.

Facilitating Web-Based Collaboration in Evidence Synthesis (TaskExchange):

Development and Analysis. JMIR Res Protoc. 2018 Dec 13;7(12):e188. doi:

10.2196/resprot.9285. PMID: 30545818; PMCID: PMC6315246.

115: Chokshi SK, Mann DM. Innovating From Within: A Process Model for User-

Centered Digital Development in Academic Medical Centers. JMIR Hum Factors. 2018

Dec 19;5(4):e11048. doi: 10.2196/11048. PMID: 30567688; PMCID: PMC6315266.

116: Pham Q, Graham G, Lalloo C, Morita PP, Seto E, Stinson JN, Cafazzo JA. An

Analytics Platform to Evaluate Effective Engagement With Pediatric Mobile Health

Apps: Design, Development, and Formative Evaluation. JMIR Mhealth Uhealth. 2018

Dec 21;6(12):e11447. doi: 10.2196/11447. PMID: 30578179; PMCID: PMC6320392.

117: Jackson S, Yaqub M, Li CX. The Agile Deployment of Machine Learning Models

in Healthcare. Front Big Data. 2019 Jan 8;1:7. doi: 10.3389/fdata.2018.00007.

PMID: 33693323; PMCID: PMC7931926.

118: Crehan C, Kesler E, Nambiar B, Dube Q, Lufesi N, Giaccone M, Normand C,

Azad K, Heys M. The NeoTree application: developing an integrated mHealth

solution to improve quality of newborn care and survival in a district hospital

in Malawi. BMJ Glob Health. 2019 Jan 16;4(1):e000860. doi:

10.1136/bmjgh-2018-000860. PMID: 30713745; PMCID: PMC6340059.

119: Maraver P, Armañanzas R, Gillette TA, Ascoli GA. PaperBot: open-source web-

based search and metadata organization of scientific literature. BMC

Bioinformatics. 2019 Jan 24;20(1):50. doi: 10.1186/s12859-019-2613-z. PMID:

30678631; PMCID: PMC6345070.

120: Cahill J, Portales R, McLoughin S, Nagan N, Henrichs B, Wetherall S.

IoT/Sensor-Based Infrastructures Promoting a Sense of Home, Independent Living,

Comfort and Wellness. Sensors (Basel). 2019 Jan 24;19(3):485. doi:

10.3390/s19030485. PMID: 30682864; PMCID: PMC6387202.

121: Cindolo L, Antonelli A, Sandri M, Annino F, Celia A, De Concilio B,

Giommoni V, Nucciotti R, Sessa F, Porreca A, Veccia A, Schips L, Minervini A;

AGILE Group (Italian Group For Advanced Laparo-Endoscopic Surgery). The role of

vascular clamping during robot-assisted partial nephrectomy for localized renal

cancer: rationale and design of the CLOCK randomized phase III study. Minerva

Urol Nefrol. 2019 Feb;71(1):96-100. doi: 10.23736/S0393-2249.18.03357-X. Epub

2019 Jan 2. PMID: 30607928.

122: Munn Z, Aromataris E, Tufanaru C, Stern C, Porritt K, Farrow J, Lockwood C,

Stephenson M, Moola S, Lizarondo L, McArthur A, Peters M, Pearson A, Jordan Z.

The development of software to support multiple systematic review types: the

Joanna Briggs Institute System for the Unified Management, Assessment and Review

of Information (JBI SUMARI). Int J Evid Based Healthc. 2019 Mar;17(1):36-43.

doi: 10.1097/XEB.0000000000000152. PMID: 30239357.

123: Mann DM, Chokshi SK, Lebwohl R, Mainiero M, Dinh-Le C, Driscoll K, Robinson

S, Egger H. Building digital innovation capacity at a large academic medical

center. NPJ Digit Med. 2019 Mar 7;2:13. doi: 10.1038/s41746-019-0088-y. PMID:

31304362; PMCID: PMC6550180.

124: Poulton A, Pan J, Bruns LR Jr, Sinnott RO, Hester R. A Smartphone App to

Assess Alcohol Consumption Behavior: Development, Compliance, and Reactivity.

JMIR Mhealth Uhealth. 2019 Mar 25;7(3):e11157. doi: 10.2196/11157. PMID:

30907738; PMCID: PMC6452287.

125: Lieu TA, Herrinton LJ, Buzkov DE, Liu L, Lyons D, Neugebauer R, Needham T,

Ng D, Prausnitz S, Stewart K, Van Den Eeden SK, Baer DM. Developing a Prognostic

Information System for Personalized Care in Real Time. EGEMS (Wash DC). 2019 Mar

25;7(1):2. doi: 10.5334/egems.266. PMID: 30937324; PMCID: PMC6437692.

126: Whiteley L, Mena L, Craker LK, Healy MG, Brown LK. Creating a Theoretically

Grounded Gaming App to Increase Adherence to Pre-Exposure Prophylaxis: Lessons

From the Development of the Viral Combat Mobile Phone Game. JMIR Serious Games.

2019 Mar 27;7(1):e11861. doi: 10.2196/11861. PMID: 30916652; PMCID: PMC6456850.

127: Neumann K, Reichl V, Rong O. Dringender Handlungsbedarf für die Zukunft des

digitalen Krankenhauses [Urgent need of action for the future of digital

hospitals]. HNO. 2019 May;67(5):350-355. German. doi: 10.1007/s00106-019-0655-1.

PMID: 30963220.

128: Jamieson T, Mamdani MM, Etchells E. Linking Quality Improvement and Health

Information Technology through the QI-HIT Figure 8. Appl Clin Inform. 2019

May;10(3):528-533. doi: 10.1055/s-0039-1693456. Epub 2019 Jul 24. PMID:

31340398; PMCID: PMC6656570.

129: Erguera XA, Johnson MO, Neilands TB, Ruel T, Berrean B, Thomas S, Saberi P.

WYZ: a pilot study protocol for designing and developing a mobile health

application for engagement in HIV care and medication adherence in youth and

young adults living with HIV. BMJ Open. 2019 May 5;9(5):e030473. doi:

10.1136/bmjopen-2019-030473. PMID: 31061063; PMCID: PMC6501960.

130: Van Camp PJ, Mahdi CM, Liu L, Ni Y, Spooner SA, Wu DTY. Development and

Preliminary Evaluation of a Visual Annotation Tool to Rapidly Collect Expert-

Annotated Weight Errors in Pediatric Growth Charts. Stud Health Technol Inform.

2019 Aug 21;264:853-857. doi: 10.3233/SHTI190344. PMID: 31438045.

131: Stanczyk FZ, Niu C, Azen C, Mirkin S, Amadio JM. Determination of estradiol

and progesterone content in capsules and creams from compounding pharmacies.

Menopause. 2019 Sep;26(9):966-971. doi: 10.1097/GME.0000000000001356. PMID:

31453957; PMCID: PMC6738624.

132: Goldstein I, Simon JA, Kaunitz AM, Altomare C, Yoshida Y, Zhu J, Schaffer

S, Soulban G. Effects of ospemifene on genitourinary health assessed by

prospective vulvar-vestibular photography and vaginal/vulvar health indices.

Menopause. 2019 Sep;26(9):994-1001. doi: 10.1097/GME.0000000000001350. PMID:

31453961; PMCID: PMC6738630.

133: Allsop MJ, Johnson O, Taylor S, Hackett J, Allen P, Bennett MI, Bewick BM.

Multidisciplinary Software Design for the Routine Monitoring and Assessment of

Pain in Palliative Care Services: The Development of PainCheck. JCO Clin Cancer

Inform. 2019 Sep;3:1-17. doi: 10.1200/CCI.18.00120. PMID: 31577449; PMCID:

PMC6873922.

134: Coppinger C, O'Loughlin R. Newborn Sickle Cell and Thalassaemia Screening

Programme: Automating and Enhancing the System to Evaluate the Screening

Programme. Int J Neonatal Screen. 2019 Aug 31;5(3):30. doi: 10.3390/ijns5030030.

PMID: 33072989; PMCID: PMC7510245.

135: Pham Q, Shaw J, Morita PP, Seto E, Stinson JN, Cafazzo JA. The Service of

Research Analytics to Optimize Digital Health Evidence Generation: Multilevel

Case Study. J Med Internet Res. 2019 Nov 11;21(11):e14849. doi: 10.2196/14849.

PMID: 31710296; PMCID: PMC6878108.

136: Appleton KM, Passmore D, Burn I, Pidgeon H, Nation P, Boobyer C, Jiang N.

An Interactive Mobile Phone App (SMART 5-A-DAY) for Increasing Knowledge of and

Adherence to Fruit and Vegetable Recommendations: Development and Pilot

Randomized Controlled Trial. JMIR Mhealth Uhealth. 2019 Nov 20;7(11):e14380.

doi: 10.2196/14380. PMID: 31746766; PMCID: PMC6893570.

137: Demirer M, Candemir S, Bigelow MT, Yu SM, Gupta V, Prevedello LM, White RD,

Yu JS, Grimmer R, Wels M, Wimmer A, Halabi AH, Ihsani A, O'Donnell TP, Erdal BS.

A User Interface for Optimizing Radiologist Engagement in Image Data Curation

for Artificial Intelligence. Radiol Artif Intell. 2019 Nov 27;1(6):e180095. doi:

10.1148/ryai.2019180095. PMID: 33937804; PMCID: PMC8017380.

138: Hales AA, Cable D, Crossley E, Findlay C, Rew DA. Design and implementation

of the stacked, synchronised and iconographic timeline-structured electronic

patient record in a UK NHS Global Digital Exemplar hospital. BMJ Health Care

Inform. 2019 Dec;26(1):e100025. doi: 10.1136/bmjhci-2019-100025. PMID: 31874854;

PMCID: PMC7252964.

139: Verhagen CD, de Boer SBB, Cardona Cano S, Vermeiren RRJM. Agile ontwikkelen

van een m-healthapp met jongeren in de ggz: een praktijk-experiment [The

development of an mHealth app with young people in mental health care using

Agile; a practical experiment]. Tijdschr Psychiatr. 2020;62(9):768-775. Dutch.

PMID: 32910448.

140: Blake H, Somerset S, Evans C. Development and Fidelity Testing of the

Test@Work Digital Toolkit for Employers on Workplace Health Checks and Opt-In

HIV Testing. Int J Environ Res Public Health. 2020 Jan 6;17(1):379. doi:

10.3390/ijerph17010379. PMID: 31935985; PMCID: PMC6982120.

141: Bricker JB, Watson NL, Heffner JL, Sullivan B, Mull K, Kwon D, Westmaas JL,

Ostroff J. A Smartphone App Designed to Help Cancer Patients Stop Smoking:

Results From a Pilot Randomized Trial on Feasibility, Acceptability, and

Effectiveness. JMIR Form Res. 2020 Jan 17;4(1):e16652. doi: 10.2196/16652. PMID:

31951215; PMCID: PMC6996729.

142: Ten Klooster I, Noordzij ML, Kelders SM. Exploring How Professionals Within

Agile Health Care Informatics Perceive Visualizations of Log File Analyses:

Observational Study Followed by a Focus Group Interview. JMIR Hum Factors. 2020

Jan 21;7(1):e14424. doi: 10.2196/14424. PMID: 31961325; PMCID: PMC7001047.

143: Kohavi R, Tang D, Xu Y, Hemkens LG, Ioannidis JPA. Online randomized

controlled experiments at scale: lessons and extensions to medicine. Trials.

2020 Feb 7;21(1):150. doi: 10.1186/s13063-020-4084-y. PMID: 32033614; PMCID:

PMC7007661.

144: Improta G, Guizzi G, Ricciardi C, Giordano V, Ponsiglione AM, Converso G,

Triassi M. Agile Six Sigma in Healthcare: Case Study at Santobono Pediatric

Hospital. Int J Environ Res Public Health. 2020 Feb 7;17(3):1052. doi:

10.3390/ijerph17031052. PMID: 32046052; PMCID: PMC7037742.

145: Gryech I, Ben-Aboud Y, Guermah B, Sbihi N, Ghogho M, Kobbane A. MoreAir: A

Low-Cost Urban Air Pollution Monitoring System. Sensors (Basel). 2020 Feb

13;20(4):998. doi: 10.3390/s20040998. PMID: 32069821; PMCID: PMC7071408.

146: Gentili C, Zetterqvist V, Rickardsson J, Holmström L, Simons LE, Wicksell

RK. ACTsmart - development and feasibility of digital Acceptance and Commitment

Therapy for adults with chronic pain. NPJ Digit Med. 2020 Feb 13;3:20. doi:

10.1038/s41746-020-0228-4. PMID: 32128450; PMCID: PMC7018849.

147: Blake H, Bermingham F, Johnson G, Tabner A. Mitigating the Psychological

Impact of COVID-19 on Healthcare Workers: A Digital Learning Package. Int J

Environ Res Public Health. 2020 Apr 26;17(9):2997. doi: 10.3390/ijerph17092997.

PMID: 32357424; PMCID: PMC7246821.

148: Moyse K, Enderby P, Chadd K, Gadhok K, Bedwell M, Guest P. Outcome

measurement in speech and language therapy: a digital journey. BMJ Health Care

Inform. 2020 May;27(1):e100085. doi: 10.1136/bmjhci-2019-100085. PMID: 32385040;

PMCID: PMC7245405.

149: Carey VJ, Ramos M, Stubbs BJ, Gopaulakrishnan S, Oh S, Turaga N, Waldron L,

Morgan M. Global Alliance for Genomics and Health Meets Bioconductor: Toward

Reproducible and Agile Cancer Genomics at Cloud Scale. JCO Clin Cancer Inform.

2020 May;4:472-479. doi: 10.1200/CCI.19.00111. PMID: 32453635; PMCID:

PMC7265787.

150: Noble L, Scott L, Stewart-Isherwood L, Molifi SJ, Sanne I, Da Silva P,

Stevens W. Continuous quality monitoring in the field: an evaluation of the

performance of the Fio Deki Reader™ for rapid HIV testing in South Africa. BMC

Infect Dis. 2020 May 4;20(1):320. doi: 10.1186/s12879-020-4932-0. PMID:

32366227; PMCID: PMC7199324.

151: Meinert E, Milne-Ives M, Surodina S, Lam C. Agile Requirements Engineering

and Software Planning for a Digital Health Platform to Engage the Effects of

Isolation Caused by Social Distancing: Case Study. JMIR Public Health Surveill.

2020 May 6;6(2):e19297. doi: 10.2196/19297. PMID: 32348293; PMCID: PMC7205031.

152: Vollmer Dahlke D, Ory MG. Emerging Issues of Intelligent Assistive

Technology Use Among People With Dementia and Their Caregivers: A U.S.

Perspective. Front Public Health. 2020 May 21;8:191. doi:

10.3389/fpubh.2020.00191. PMID: 32528920; PMCID: PMC7254691.

153: Voigt I, Benedict M, Susky M, Scheplitz T, Frankowitz S, Kern R, Müller O,

Schlieter H, Ziemssen T. A Digital Patient Portal for Patients With Multiple

Sclerosis. Front Neurol. 2020 May 22;11:400. doi: 10.3389/fneur.2020.00400.

PMID: 32670174; PMCID: PMC7326091.

154: Schinköthe T, Gabri MR, Mitterer M, Gouveia P, Heinemann V, Harbeck N,

Subklewe M. A Web- and App-Based Connected Care Solution for COVID-19 In- and

Outpatient Care: Qualitative Study and Application Development. JMIR Public

Health Surveill. 2020 Jun 1;6(2):e19033. doi: 10.2196/19033. PMID: 32406855;

PMCID: PMC7265653.

155: Griffiths G, Fitzgerald R, Jaki T, Corkhill A, Marwood E, Reynolds H,

Stanton L, Ewings S, Condie S, Wrixon E, Norton A, Radford M, Yeats S, Robertson

J, Darby-Dowman R, Walker L, Khoo S; UK NIHR community. AGILE-ACCORD: A

Randomized, Multicentre, Seamless, Adaptive Phase I/II Platform Study to

Determine the Optimal Dose, Safety and Efficacy of Multiple Candidate Agents for

the Treatment of COVID-19: A structured summary of a study protocol for a

randomised platform trial. Trials. 2020 Jun 19;21(1):544. doi:

10.1186/s13063-020-04473-1. PMID: 32560744; PMCID: PMC7303573.

156: Taylor HA Jr, Francis S, Evans CR, Harvey M, Newton BA, Jones CP, Akintobi

TH, Clifford G. Preventing Cardiovascular Disease Among Urban African Americans

With a Mobile Health App (the MOYO App): Protocol for a Usability Study. JMIR

Res Protoc. 2020 Jul 9;9(7):e16699. doi: 10.2196/16699. PMID: 32673258; PMCID:

PMC7380980.

157: Navarro-Alamán J, Lacuesta R, García-Magariño I, Gallardo J, Ibarz E,

Lloret J. Close2U: An App for Monitoring Cancer Patients with Enriched

Information from Interaction Patterns. J Healthc Eng. 2020 Jul 15;2020:3057032.

doi: 10.1155/2020/3057032. PMID: 32733661; PMCID: PMC7378616.

158: Spengler H, Lang C, Mahapatra T, Gatz I, Kuhn KA, Prasser F. Enabling Agile

Clinical and Translational Data Warehousing: Platform Development and

Evaluation. JMIR Med Inform. 2020 Jul 21;8(7):e15918. doi: 10.2196/15918. PMID:

32706673; PMCID: PMC7404007.

159: Lepperød ME, Dragly SA, Buccino AP, Mobarhan MH, Malthe-Sørenssen A,

Hafting T, Fyhn M. Experimental Pipeline (Expipe): A Lightweight Data Management

Platform to Simplify the Steps From Experiment to Data Analysis. Front

Neuroinform. 2020 Jul 24;14:30. doi: 10.3389/fninf.2020.00030. PMID: 32792932;

PMCID: PMC7393253.

160: Andrade PHS, de Almeida ACB, Dos Santos AKS, Lobo IMF, da Silva FA, da

Silva WB. Challenges to the consolidation of pharmacovigilance practices in

Brazil: limitations of the hospital pharmacist. Ther Adv Drug Saf. 2020 Jul

31;11:2042098620933748. doi: 10.1177/2042098620933748. PMID: 32864089; PMCID:

PMC7430076.

161: Weemaes M, Martens S, Cuypers L, Van Elslande J, Hoet K, Welkenhuysen J,

Goossens R, Wouters S, Houben E, Jeuris K, Laenen L, Bruyninckx K, Beuselinck K,

André E, Depypere M, Desmet S, Lagrou K, Van Ranst M, Verdonck AKLC, Goveia J.

Laboratory information system requirements to manage the COVID-19 pandemic: A

report from the Belgian national reference testing center. J Am Med Inform

Assoc. 2020 Aug 1;27(8):1293-1299. doi: 10.1093/jamia/ocaa081. PMID: 32348469;

PMCID: PMC7197526.

162: Baker D, van den Beek M, Blankenberg D, Bouvier D, Chilton J, Coraor N,

Coppens F, Eguinoa I, Gladman S, Grüning B, Keener N, Larivière D, Lonie A,

Kosakovsky Pond S, Maier W, Nekrutenko A, Taylor J, Weaver S. No more business

as usual: Agile and effective responses to emerging pathogen threats require

open data and open analytics. PLoS Pathog. 2020 Aug 13;16(8):e1008643. doi:

10.1371/journal.ppat.1008643. PMID: 32790776; PMCID: PMC7425854.

163: Tobias G, Spanier AB. Developing a Mobile App (iGAM) to Promote Gingival

Health by Professional Monitoring of Dental Selfies: User-Centered Design

Approach. JMIR Mhealth Uhealth. 2020 Aug 14;8(8):e19433. doi: 10.2196/19433.

PMID: 32795985; PMCID: PMC7455872.

164: Allen WE, Altae-Tran H, Briggs J, Jin X, McGee G, Shi A, Raghavan R,

Kamariza M, Nova N, Pereta A, Danford C, Kamel A, Gothe P, Milam E, Aurambault

J, Primke T, Li W, Inkenbrandt J, Huynh T, Chen E, Lee C, Croatto M, Bentley H,

Lu W, Murray R, Travassos M, Coull BA, Openshaw J, Greene CS, Shalem O, King G,

Probasco R, Cheng DR, Silbermann B, Zhang F, Lin X. Population-scale

longitudinal mapping of COVID-19 symptoms, behaviour and testing. Nat Hum Behav.

2020 Sep;4(9):972-982. doi: 10.1038/s41562-020-00944-2. Epub 2020 Aug 26. PMID:

32848231; PMCID: PMC7501153.

165: Bhatia V, Mandal PP, Satyanarayana S, Aditama TY, Sharma M. Mitigating the

impact of the COVID-19 pandemic on progress towards ending tuberculosis in the

WHO South-East Asia Region. WHO South East Asia J Public Health. 2020

Sep;9(2):95-99. doi: 10.4103/2224-3151.294300. PMID: 32978339.

166: Ribaut J, Leppla L, Teynor A, Valenta S, Dobbels F, Zullig LL, De Geest S;

SMILe study team. Theory-driven development of a medication adherence

intervention delivered by eHealth and transplant team in allogeneic stem cell

transplantation: the SMILe implementation science project. BMC Health Serv Res.

2020 Sep 2;20(1):827. doi: 10.1186/s12913-020-05636-1. PMID: 32878623; PMCID:

PMC7465386.

167: Backman C, Harley A, Kuziemsky C, Mercer J, Peyton L. MyPath to Home Web-

Based Application for the Geriatric Rehabilitation Program at Bruyère Continuing

Care: User-Centered Design and Feasibility Testing Study. JMIR Form Res. 2020

Sep 14;4(9):e18169. doi: 10.2196/18169. PMID: 32924953; PMCID: PMC7522728.

168: Zotov E, Hills AF, de Mello FL, Aram P, Sayers A, Blom AW, McCloskey EV,

Wilkinson JM, Kadirkamanathan V. JointCalc: A web-based personalised patient

decision support tool for joint replacement. Int J Med Inform. 2020

Oct;142:104217. doi: 10.1016/j.ijmedinf.2020.104217. Epub 2020 Jun 25. PMID:

32853974; PMCID: PMC7607377.

169: Haddad SM, Souza RT, Cecatti JG, Barreix M, Tamrat T, Footitt C, Mehl GL,

Syah IF, Shankar AH, Tunçalp Ö. Building a Digital Tool for the Adoption of the

World Health Organization's Antenatal Care Recommendations: Methodological

Intersection of Evidence, Clinical Logic, and Digital Technology. J Med Internet

Res. 2020 Oct 1;22(10):e16355. doi: 10.2196/16355. Erratum in: J Med Internet

Res. 2020 Oct 13;22(10):e24891. PMID: 33001032; PMCID: PMC7983224.

170: Chen Y, Banerjee A. Improving the digital health of the workforce in the

COVID-19 context: an opportunity to future-proof medical training. Future

Healthc J. 2020 Oct;7(3):189-192. doi: 10.7861/fhj.2020-0162. PMID: 33094221;

PMCID: PMC7571768.

171: Zhang H, Dimitrov D, Simpson L, Plaks N, Singh B, Penney S, Charles J,

Sheehan R, Flammini S, Murphy S, Landman A. A Web-Based, Mobile-Responsive

Application to Screen Health Care Workers for COVID-19 Symptoms: Rapid Design,

Deployment, and Usage. JMIR Form Res. 2020 Oct 8;4(10):e19533. doi:

10.2196/19533. PMID: 32877348; PMCID: PMC7546861.

172: Narla NP, Surmeli A, Kivlehan SM. Agile Application of Digital Health

Interventions during the COVID-19 Refugee Response. Ann Glob Health. 2020 Oct

15;86(1):135. doi: 10.5334/aogh.2995. PMID: 33117656; PMCID: PMC7566526.

173: Mehta J, Yates T, Smith P, Henderson D, Winteringham G, Burns A. Rapid

implementation of Microsoft Teams in response to COVID-19: one acute healthcare

organisation's experience. BMJ Health Care Inform. 2020 Nov;27(3):e100209. doi:

10.1136/bmjhci-2020-100209. PMID: 33177050; PMCID: PMC7661347.

174: Naik SS, Manjunatha N, Kumar CN, Math SB, Moirangthem S. Patient's

Perspectives of Telepsychiatry: The Past, Present and Future. Indian J Psychol

Med. 2020 Nov 1;42(5 Suppl):102S-107S. doi: 10.1177/0253717620963341. PMID:

33354053; PMCID: PMC7736740.

175: Dhahri AA, Iqbal MR, Pardoe H. Agile Application of Video Telemedicine

During the COVID-19 Pandemic. Cureus. 2020 Nov 4;12(11):e11320. doi:

10.7759/cureus.11320. PMID: 33262918; PMCID: PMC7689966.

176: Marston HR, Shore L, White PJ. How does a (Smart) Age-Friendly Ecosystem

Look in a Post-Pandemic Society? Int J Environ Res Public Health. 2020 Nov

9;17(21):8276. doi: 10.3390/ijerph17218276. PMID: 33182413; PMCID: PMC7664882.

177: Miah SJ, Gammack J, Hasan N. Methodologies for designing healthcare

analytics solutions: A literature analysis. Health Informatics J. 2020

Dec;26(4):2300-2314. doi: 10.1177/1460458219895386. Epub 2019 Dec 26. PMID:

31876227.

178: Murphy BP, O'Raghallaigh P, Carr M. Nurturing the digital baby: Open

innovation for development and optimization. Health Informatics J. 2020

Dec;26(4):2407-2421. doi: 10.1177/1460458220906067. Epub 2020 Feb 25. PMID:

32098558.

179: Lei H, O'Connell R, Ehwerhemuepha L, Taraman S, Feaster W, Chang A. Agile

clinical research: A data science approach to scrumban in clinical medicine.

Intell Based Med. 2020 Dec;3:100009. doi: 10.1016/j.ibmed.2020.100009. Epub 2020

Oct 22. PMID: 33106798; PMCID: PMC7578702.

180: Jalloh MF, Sengeh P, James N, Bah S, Jalloh MB, Owen K, Pratt SA, Oniba A,

Sangarie M, Sesay S, Bedson J. Integrated digital system for community

engagement and community-based surveillance during the 2014-2016 Ebola outbreak

in Sierra Leone: lessons for future health emergencies. BMJ Glob Health. 2020

Dec;5(12):e003936. doi: 10.1136/bmjgh-2020-003936. PMID: 33355270; PMCID:

PMC7757454.

181: Lam C, van Velthoven MH, Meinert E. Developing a Blockchain-Based Supply

Chain System for Advanced Therapies: Protocol for a Feasibility Study. JMIR Res

Protoc. 2020 Dec 14;9(12):e17005. doi: 10.2196/17005. PMID: 33315020; PMCID:

PMC7769686.

182: Mello LRG, Christovam BP, Moreira APA, Moraes EB. Safety Huddle methodology

development in patient safety software: an experience report. Rev Bras Enferm.

2020 Dec 21;73(suppl 6):e20190788. English, Portuguese. doi:

10.1590/0034-7167-2019-0788. PMID: 33338149.

183: Gaspar JS, Lage EM, Silva FJD, Mineiro É, Oliveira IJR, Oliveira I, Souza

RG, Gusmão JRO, De Souza CFD, Reis ZSN. A Mobile Serious Game About the Pandemic

(COVID-19 - Did You Know?): Design and Evaluation Study. JMIR Serious Games.

2020 Dec 22;8(4):e25226. doi: 10.2196/25226. PMID: 33301416; PMCID: PMC7758085.

184: Baldasso RP, Moraes C, Gallardo E, Stumvoll MB, Crespo KC, Strapasson RAP,

de Oliveira RN. 3D forensic facial approximation: Implementation protocol in a

forensic activity. J Forensic Sci. 2021 Jan;66(1):383-388. doi:

10.1111/1556-4029.14587. Epub 2020 Oct 7. PMID: 33027540.

185: Varsavsky T, Graham MS, Canas LS, Ganesh S, Capdevila Pujol J, Sudre CH,

Murray B, Modat M, Jorge Cardoso M, Astley CM, Drew DA, Nguyen LH, Fall T, Gomez

MF, Franks PW, Chan AT, Davies R, Wolf J, Steves CJ, Spector TD, Ourselin S.

Detecting COVID-19 infection hotspots in England using large-scale self-reported

data from a mobile application: a prospective, observational study. Lancet

Public Health. 2021 Jan;6(1):e21-e29. doi: 10.1016/S2468-2667(20)30269-3. Epub

2020 Dec 3. PMID: 33278917; PMCID: PMC7785969.

186: Leppla L, Hobelsberger S, Rockstein D, Werlitz V, Pschenitza S, Heidegger

P, De Geest S, Valenta S, Teynor A; SMILe study team. Implementation Science

Meets Software Development to Create eHealth Components for an Integrated Care

Model for Allogeneic Stem Cell Transplantation Facilitated by eHealth: The SMILe

Study as an Example. J Nurs Scholarsh. 2021 Jan;53(1):35-45. doi:

10.1111/jnu.12621. Epub 2020 Dec 21. PMID: 33348461.

187: Bolislis WR, de Lucia ML, Dolz F, Mo R, Nagaoka M, Rodriguez H, Woon ML, Yu

W, Kühler TC. Regulatory Agilities in the Time of COVID-19: Overview, Trends,

and Opportunities. Clin Ther. 2021 Jan;43(1):124-139. doi:

10.1016/j.clinthera.2020.11.015. Epub 2020 Nov 30. PMID: 33353762; PMCID:

PMC7703519.

188: Wacksman J. Digitalization of contact tracing: balancing data privacy with

public health benefit. Ethics Inf Technol. 2021;23(4):855-861. doi:

10.1007/s10676-021-09601-2. Epub 2021 Jun 10. PMID: 34131391; PMCID: PMC8192038.

189: Mao Z, Yao H, Zou Q, Zhang W, Dong Y. Digital Contact Tracing Based on a

Graph Database Algorithm for Emergency Management During the COVID-19 Epidemic:

Case Study. JMIR Mhealth Uhealth. 2021 Jan 22;9(1):e26836. doi: 10.2196/26836.

PMID: 33460389; PMCID: PMC7837510.

190: Pach D, Rogge AA, Wang J, Witt CM. Five Lessons Learned From Randomized

Controlled Trials on Mobile Health Interventions: Consensus Procedure on

Practical Recommendations for Sustainable Research. JMIR Mhealth Uhealth. 2021

Feb 8;9(2):e20630. doi: 10.2196/20630. PMID: 33555263; PMCID: PMC7899803.

191: Lawrence K, Rodriguez DV, Feldthouse DM, Shelley D, Yu JL, Belli HM,

Gonzalez J, Tasneem S, Fontaine J, Groom LL, Luu S, Wu Y, McTigue KM, Rockette-

Wagner B, Mann DM. Effectiveness of an Integrated Engagement Support System to

Facilitate Patient Use of Digital Diabetes Prevention Programs: Protocol for a

Randomized Controlled Trial. JMIR Res Protoc. 2021 Feb 9;10(2):e26750. doi:

10.2196/26750. PMID: 33560240; PMCID: PMC7902197.

192: Rubiano L, Alexander NDE, Castillo RM, Martínez ÁJ, García Luna JA, Arango

JD, Vargas L, Madriñán P, Hurtado LR, Orobio Y, Rojas CA, Del Corral H, Navarro

A, Gore Saravia N, Aronoff-Spencer E. Adaptation and performance of a mobile

application for early detection of cutaneous leishmaniasis. PLoS Negl Trop Dis.

2021 Feb 11;15(2):e0008989. doi: 10.1371/journal.pntd.0008989. PMID: 33571192;

PMCID: PMC7904137.

193: Jeyakumar T, McClure S, Lowe M, Hodges B, Fur K, Javier-Brozo M, Tassone M,

Anderson M, Tripp T, Wiljer D. An Education Framework for Effective

Implementation of a Health Information System: Scoping Review. J Med Internet

Res. 2021 Feb 24;23(2):e24691. doi: 10.2196/24691. PMID: 33625370; PMCID:

PMC7946593.

194: Lim HM, Teo CH, Ng CJ, Chiew TK, Ng WL, Abdullah A, Abdul Hadi H, Liew CS,

Chan CS. An Automated Patient Self-Monitoring System to Reduce Health Care

System Burden During the COVID-19 Pandemic in Malaysia: Development and

Implementation Study. JMIR Med Inform. 2021 Feb 26;9(2):e23427. doi:

10.2196/23427. PMID: 33600345; PMCID: PMC7919845.

195: Arabi YM, Azoulay E, Al-Dorzi HM, Phua J, Salluh J, Binnie A, Hodgson C,

Angus DC, Cecconi M, Du B, Fowler R, Gomersall CD, Horby P, Juffermans NP,

Kesecioglu J, Kleinpell RM, Machado FR, Martin GS, Meyfroidt G, Rhodes A, Rowan

K, Timsit JF, Vincent JL, Citerio G. How the COVID-19 pandemic will change the

future of critical care. Intensive Care Med. 2021 Mar;47(3):282-291. doi:

10.1007/s00134-021-06352-y. Epub 2021 Feb 22. PMID: 33616696; PMCID: PMC7898492.

196: Sullivan C, Wong I, Adams E, Fahim M, Fraser J, Ranatunga G, Busato M,

McNeil K. Moving Faster than the COVID-19 Pandemic: The Rapid, Digital

Transformation of a Public Health System. Appl Clin Inform. 2021

Mar;12(2):229-236. doi: 10.1055/s-0041-1725186. Epub 2021 Mar 24. PMID:

33763847; PMCID: PMC7990571.

197: Dubuc N, Brière S, Corbin C, N'Bouke A, Bonin L, Delli-Colli N.

Computerized Care-Pathways (CCPs) System to Support Person-Centered, Integrated,

and Proactive Care in Home-Care Settings. Inform Health Soc Care. 2021 Mar

2;46(1):100-111. doi: 10.1080/17538157.2020.1865969. Epub 2021 Jan 6. PMID:

33406972.

198: Hill JR, Harrington AB, Adeoye P, Campbell NL, Holden RJ. Going Remote-

Demonstration and Evaluation of Remote Technology Delivery and Usability

Assessment With Older Adults: Survey Study. JMIR Mhealth Uhealth. 2021 Mar

4;9(3):e26702. doi: 10.2196/26702. PMID: 33606655; PMCID: PMC7935399.

199: Gárate FJ, Chausa P, Whetham J, Jones CI, García F, Cáceres C, Sánchez-

González P, Wallitt E, Gómez EJ, On Behalf Of The EmERGE Consortium. EmERGE

mHealth Platform: Implementation and Technical Evaluation of a Digital Supported

Pathway of Care for Medically Stable HIV. Int J Environ Res Public Health. 2021

Mar 18;18(6):3156. doi: 10.3390/ijerph18063156. PMID: 33803821; PMCID:

PMC8003226.

200: Harshbarger C, Burrus O, Rangarajan S, Bollenbacher J, Zulkiewicz B, Verma

R, Galindo CA, Lewis MA. Challenges of and Solutions for Developing Tailored

Video Interventions That Integrate Multiple Digital Assets to Promote Engagement

and Improve Health Outcomes: Tutorial. JMIR Mhealth Uhealth. 2021 Mar

23;9(3):e21128. doi: 10.2196/21128. PMID: 33755025; PMCID: PMC8294466.

201: Berlin A, Lovas M, Truong T, Melwani S, Liu J, Liu ZA, Badzynski A,

Carpenter MB, Virtanen C, Morley L, Bhattacharyya O, Escaf M, Moody L, Goldfarb

A, Brzozowski L, Cafazzo J, Chua MLK, Stewart AK, Krzyzanowska MK.

Implementation and Outcomes of Virtual Care Across a Tertiary Cancer Center

During COVID-19. JAMA Oncol. 2021 Apr 1;7(4):597-602. doi:

10.1001/jamaoncol.2020.6982. PMID: 33410867; PMCID: PMC7791400.

202: Elsworth B, Gaunt TR. MELODI Presto: a fast and agile tool to explore

semantic triples derived from biomedical literature. Bioinformatics. 2021 May

1;37(4):583-585. doi: 10.1093/bioinformatics/btaa726. PMID: 32810207; PMCID:

PMC8088324.

203: Lo WC, Wang FC, Lin LY, Jyan HW, Wu HC, Huang YL, Parng IM, Chiou HY.

Enhancing Data Linkage to Break the Chain of COVID-19 Spread: The Taiwan

Experience. J Med Internet Res. 2021 May 7;23(5):e24294. doi: 10.2196/24294.

PMID: 33882019; PMCID: PMC8108927.

204: Odeh M, Kharbat FF, Yousef R, Odeh Y, Tbaishat D, Hakooz N, Dajani R,

Mansour A. iOntoBioethics: A Framework for the Agile Development of Bioethics

Ontologies in Pandemics, Applied to COVID-19. Front Med (Lausanne). 2021 May

21;8:619978. doi: 10.3389/fmed.2021.619978. PMID: 34095160; PMCID: PMC8175792.

205: Jake-Schoffman DE, McVay MA. Using the Design Sprint process to enhance and

accelerate behavioral medicine progress: a case study and guidance. Transl Behav

Med. 2021 May 25;11(5):1099-1106. doi: 10.1093/tbm/ibaa100. PMID: 33057685.

206: Impouma B, Mlanda T, Bukhari A, Sie Williams G, Farham B, Wolfe C, Mboussou

F, Botero Mesa S, Ngom R, Lee T, Keiser O. Information management practices in

the WHO African Region to support response to the COVID-19 pandemic. Epidemiol

Infect. 2021 May 26;149:e260. doi: 10.1017/S0950268821001242. PMID: 34036928;

PMCID: PMC8712935.

207: Tanniru MR, Agarwal N, Sokan A, Hariri S. An Agile Digital Platform to

Support Population Health-A Case Study of a Digital Platform to Support Patients

with Delirium Using IoT, NLP, and AI. Int J Environ Res Public Health. 2021 May

26;18(11):5686. doi: 10.3390/ijerph18115686. PMID: 34073262; PMCID: PMC8198835.

208: Lin PF, Naveed H, Eleftheriadou M, Purbrick R, Zarei Ghanavati M, Liu C.

Cataract service redesign in the post-COVID-19 era. Br J Ophthalmol. 2021

Jun;105(6):745-750. doi: 10.1136/bjophthalmol-2020-316917. Epub 2020 Jul 23.

PMID: 32703783.

209: Finn E, Kuusinen J. Innovation Through Universal Design in Agile UX

Software Development Teams. A Collaborative Case Study of an Under Graduate AR

Tourist Guide Project. Stud Health Technol Inform. 2021 Jun 4;282:252-258. doi:

10.3233/SHTI210401. PMID: 34085973.

210: Casado J, Lehtonen O, Rantanen V, Kaipio K, Pasquini L, Häkkinen A,

Petrucci E, Hynninen J, Hietanen S, Carpén O, Biffoni M, Färkkilä A, Hautaniemi

S. Agile workflow for interactive analysis of mass cytometry data.

Bioinformatics. 2021 Jun 9;37(9):1263-1268. doi: 10.1093/bioinformatics/btaa946.

PMID: 33135052; PMCID: PMC8189671.

211: Beilharz F, Sukunesan S, Rossell SL, Kulkarni J, Sharp G. Development of a

Positive Body Image Chatbot (KIT) With Young People and Parents/Carers:

Qualitative Focus Group Study. J Med Internet Res. 2021 Jun 16;23(6):e27807.

doi: 10.2196/27807. PMID: 34132644; PMCID: PMC8277317.

212: Unsworth H, Dillon B, Collinson L, Powell H, Salmon M, Oladapo T, Ayiku L,

Shield G, Holden J, Patel N, Campbell M, Greaves F, Joshi I, Powell J, Tonnel A.

The NICE Evidence Standards Framework for digital health and care technologies -

Developing and maintaining an innovative evidence framework with global impact.

Digit Health. 2021 Jun 24;7:20552076211018617. doi: 10.1177/20552076211018617.

PMID: 34249371; PMCID: PMC8236783.

213: Herrmann S, Power B, Rashidi A, Cypher M, Mastaglia F, Grace A, McKinnon E,

Sarrot P, Michau C, Skinner M, Desai R, Duracinsky M. Supporting Patient-

Clinician Interaction in Chronic HIV Care: Design and Development of a Patient-

Reported Outcomes Software Application. J Med Internet Res. 2021 Jul

30;23(7):e27861. doi: 10.2196/27861. PMID: 34328442; PMCID: PMC8367117.

214: Behne A, Krüger N, Beinke JH, Teuteberg F. Learnings from the design and

acceptance of the German COVID-19 tracing app for IS-driven crisis management: a

design science research. BMC Med Inform Decis Mak. 2021 Aug 9;21(1):238. doi:

10.1186/s12911-021-01579-7. PMID: 34372840; PMCID: PMC8350273.

215: Ranney ML, Pittman SK, Moseley I, Morgan KE, Riese A, Ybarra M, Cunningham

R, Rosen R. Cyberbullying Prevention for Adolescents: Iterative Qualitative

Methods for Mobile Intervention Design. JMIR Form Res. 2021 Aug 27;5(8):e25900.

doi: 10.2196/25900. PMID: 34448702; PMCID: PMC8433933.

216: Lopez Segui F, Hernandez Guillamet G, Pifarré Arolas H, Marin-Gomez FX,

Ruiz Comellas A, Ramirez Morros AM, Adroher Mas C, Vidal-Alaball J.

Characterization and Identification of Variations in Types of Primary Care

Visits Before and During the COVID-19 Pandemic in Catalonia: Big Data Analysis

Study. J Med Internet Res. 2021 Sep 14;23(9):e29622. doi: 10.2196/29622. PMID:

34313600; PMCID: PMC8767991.

217: Losana P, Castro JW, Ferre X, Villalba-Mora E, Acuña ST. A Systematic

Mapping Study on Integration Proposals of the Personas Technique in Agile

Methodologies. Sensors (Basel). 2021 Sep 20;21(18):6298. doi: 10.3390/s21186298.

PMID: 34577504; PMCID: PMC8473179.

218: Schüttler C, Prokosch HU, Hummel M, Lablans M, Kroll B, Engels C; German

Biobank Alliance IT development team. The journey to establishing an IT-

infrastructure within the German Biobank Alliance. PLoS One. 2021 Sep

22;16(9):e0257632. doi: 10.1371/journal.pone.0257632. PMID: 34551019; PMCID:

PMC8457464.

219: Hensel KO, Powell J. Viewpoint: digital paediatrics-so close yet so far

away. Arch Dis Child. 2021 Sep 29:archdischild-2021-322719. doi:

10.1136/archdischild-2021-322719. Epub ahead of print. PMID: 34588169.

220: Azizoddin DR, Adam R, Kessler D, Wright AA, Kematick B, Sullivan C, Zhang

H, Hassett MJ, Cooley ME, Ehrlich O, Enzinger AC. Leveraging mobile health

technology and research methodology to optimize patient education and self-

management support for advanced cancer pain. Support Care Cancer. 2021

Oct;29(10):5741-5751. doi: 10.1007/s00520-021-06146-4. Epub 2021 Mar 18. PMID:

33738594; PMCID: PMC8410657.

221: Wildman MJ, O’Cathain A, Hind D, Maguire C, Arden MA, Hutchings M, Bradley

J, Walters SJ, Whelan P, Ainsworth J, Tappenden P, Buchan I, Elliott R, Nicholl

J, Elborn S, Michie S, Mandefield L, Sutton L, Hoo ZH, Drabble SJ, Lumley E,

Beever D, Navega Biz A, Scott A, Waterhouse S, Robinson L, Hernández Alava M,

Sasso A. An intervention to support adherence to inhaled medication in adults

with cystic fibrosis: the ACtiF research programme including RCT. Southampton

(UK): NIHR Journals Library; 2021 Oct. PMID: 34665573.

222: Soegaard Ballester JM, Bass GD, Urbani R, Fala G, Patel R, Leri D,

Steinkamp JM, Denson JL, Rosin R, Adusumalli S, Hanson CW, Koppel R, Airan-Javia

S. A Mobile, Electronic Health Record-Connected Application for Managing Team

Workflows in Inpatient Care. Appl Clin Inform. 2021 Oct;12(5):1120-1134. doi:

10.1055/s-0041-1740256. Epub 2021 Dec 22. PMID: 34937103; PMCID: PMC8695057.

223: Puli L, Layton N, Mont D, Shae K, Calvo I, Hill KD, Callaway L, Tebbutt E,

Manlapaz A, Groenewegen I, Hiscock D. Assistive Technology Provider Experiences

during the COVID-19 Pandemic. Int J Environ Res Public Health. 2021 Oct

6;18(19):10477. doi: 10.3390/ijerph181910477. PMID: 34639777; PMCID: PMC8507834.

224: Keenoy KE, Lenze EJ, Nicol GE. Going remote: Implementing digital research

methods at an academic medical center during COVID-19. J Clin Transl Sci. 2021

Oct 6;5(1):e189. doi: 10.1017/cts.2021.865. PMID: 34812289; PMCID: PMC8593367.

225: Silva MLBD, Durovini P, Mota P, Kritski AL. Fatores associados à

subnotificação de casos de tuberculose multirresistente no Estado do Rio de

Janeiro, Brasil: relacionamento probabilístico entre sistemas de informação

[Factors associated with underreporting of cases of multidrug-resistant

tuberculosis in the state of Rio de Janeiro, Brazil: probabilistic database

linkage]. Cad Saude Publica. 2021 Oct 8;37(10):e00293920. Portuguese. doi:

10.1590/0102-311X00293920. PMID: 34644761.

226: Barr PJ, Haslett W, Dannenberg MD, Oh L, Elwyn G, Hassanpour S, Bonasia KL,

Finora JC, Schoonmaker JA, Onsando WM, Ryan J, Bruce ML, Das AK, Arend R, Piper

S, Ganoe CH. An Audio Personal Health Library of Clinic Visit Recordings for

Patients and Their Caregivers (HealthPAL): User-Centered Design Approach. J Med

Internet Res. 2021 Oct 22;23(10):e25512. doi: 10.2196/25512. PMID: 34677131;

PMCID: PMC8727051.

227: Abedian S, Kolivand P, Lornejad HR. Toward an Agile System: Iranian

Information System for Covid-19-Affected Patients Data Collection from Iranian

Hospitals. Stud Health Technol Inform. 2021 Oct 27;285:173-178. doi:

10.3233/SHTI210593. PMID: 34734870.

228: Posever N, Sehdev M, Sylla M, Mashar R, Mashar M, Abioye A. Addressing

Equity in Global Medical Education During the COVID-19 Pandemic: The Global

Medical Education Collaborative. Acad Med. 2021 Nov 1;96(11):1574-1579. doi:

10.1097/ACM.0000000000004230. PMID: 34261867; PMCID: PMC8541891.

229: Pakarinen T, Ojala J. Profeel-An open source dosimetry data visualization

and analysis software. Comput Methods Programs Biomed. 2021 Nov;212:106457. doi:

10.1016/j.cmpb.2021.106457. Epub 2021 Oct 8. PMID: 34666203.

230: Gagnon Shaigetz V, Proulx C, Cabral A, Choudhury N, Hewko M, Kohlenberg E,

Segado M, Smith MSD, Debergue P. An Immersive and Interactive Platform for

Cognitive Assessment and Rehabilitation (bWell): Design and Iterative

Development Process. JMIR Rehabil Assist Technol. 2021 Nov 3;8(4):e26629. doi:

10.2196/26629. PMID: 34730536; PMCID: PMC8600432.

231: Priya B, Malhotra J. 5GhNet: an intelligent QoE aware RAT selection

framework for 5G-enabled healthcare network. J Ambient Intell Humaniz Comput.

2021 Nov 26:1-22. doi: 10.1007/s12652-021-03606-x. Epub ahead of print. PMID:

34849173; PMCID: PMC8617375.

232: Lavie G, Weinstein O, Segal Y, Davidson E. Adapting to change: Clalit's

response to the COVID-19 pandemic. Isr J Health Policy Res. 2021 Nov

30;10(1):68. doi: 10.1186/s13584-021-00498-2. PMID: 34847927; PMCID: PMC8630513.

233: Leppla L, Schmid A, Valenta S, Mielke J, Beckmann S, Ribaut J, Teynor A,

Dobbels F, Duerinckx N, Zeiser R, Engelhardt M, Gerull S, De Geest S; SMILe

study team. Development of an integrated model of care for allogeneic stem cell

transplantation facilitated by eHealth-the SMILe study. Support Care Cancer.

2021 Dec;29(12):8045-8057. doi: 10.1007/s00520-021-06328-0. Epub 2021 Jul 5.

PMID: 34224016; PMCID: PMC8550349.

234: Loo Gee B, Batterham PJ, Gulliver A, Reynolds J, Griffiths KM. An

Ecological Momentary Intervention for people with social anxiety: A descriptive

case study. Inform Health Soc Care. 2021 Dec 2;46(4):370-398. doi:

10.1080/17538157.2021.1896525. Epub 2021 Mar 28. PMID: 33779480.

235: Fox K, Price S, Achenbach S, Aguiar C, Bruining N, Cowie M, Plummer C,

Roffi M, Westwood M. The European Society of Cardiology - A Digital Educator. J

Eur CME. 2021 Dec 9;10(1):2014039. doi: 10.1080/21614083.2021.2014039. PMID:

34912587; PMCID: PMC8667914.

236: Torrente G, de Souza TQ, Tonaki L, Cardoso AP, Manickchand Junior L, da

Silva GO. Scrum Framework and Health Solutions: Management and Results. Stud

Health Technol Inform. 2021 Dec 15;284:290-294. doi: 10.3233/SHTI210725. PMID:

34920528.

237: Cooray N, Sun SL, Ho C, Adams S, Keay L, Nassar N, Brown J. Toward a

Behavior Theory-Informed and User-Centered Mobile App for Parents to Prevent

Infant Falls: Development and Usability Study. JMIR Pediatr Parent. 2021 Dec

20;4(4):e29731. doi: 10.2196/29731. PMID: 34932004; PMCID: PMC8726019.

238: Klemme I, Richter B, De Sabbata K, Wrede B, Vollmer AL. A Multi-Directional

and Agile Academic Knowledge Transfer Strategy for Healthcare Technology. Front

Robot AI. 2021 Dec 21;8:789827. doi: 10.3389/frobt.2021.789827. PMID: 34993238;

PMCID: PMC8724569.

239: Blake H, Somerset S, Greaves S. The Pain at Work Toolkit for Employees with

Chronic or Persistent Pain: A Collaborative-Participatory Study. Healthcare

(Basel). 2021 Dec 29;10(1):56. doi: 10.3390/healthcare10010056. PMID: 35052220;

PMCID: PMC8775489.

240: Silva G, Bourne T, Hall G, Patel S, Rauf MQ, Vogel A, Carruthers A, Xu G.

Codeveloping an effective EMPA to maturity in an acute NHS Trust: an implementer

report. BMJ Health Care Inform. 2022 Jan;29(1):e100477. doi:

10.1136/bmjhci-2021-100477. PMID: 34983793; PMCID: PMC8728440.

241: Hassett MJ, Cronin C, Tsou TC, Wedge J, Bian J, Dizon DS, Hazard-Jenkins H,

Osarogiagbon RU, Wong S, Basch E, Austin T, McCleary N, Schrag D. eSyM: An

Electronic Health Record-Integrated Patient-Reported Outcomes-Based Cancer

Symptom Management Program Used by Six Diverse Health Systems. JCO Clin Cancer

Inform. 2022 Jan;6:e2100137. doi: 10.1200/CCI.21.00137. PMID: 34985914.

242: Rao N, Perdomo S, Jonassaint C. A Novel Method for Digital Pain Assessment

Using Abstract Animations: Human-Centered Design Approach. JMIR Hum Factors.

2022 Jan 7;9(1):e27689. doi: 10.2196/27689. PMID: 34994697; PMCID: PMC8783278.

243: Wanyama C, Nagraj S, Muinga N, Tuti T, Edgcombe H, Geniets A, Winters N,

English M, Rossner J, Paton C. Lessons from the design, development and

implementation of a three-dimensional (3D) neonatal resuscitation training

smartphone application: Life-saving Instruction for Emergencies (LIFE app). Adv

Simul (Lond). 2022 Jan 10;7(1):2. doi: 10.1186/s41077-021-00197-7. PMID:

35012665; PMCID: PMC8744048.

244: Clifford G, Nguyen T, Shaw C, Newton B, Francis S, Salari M, Evans C, Jones

C, Akintobi TH, Taylor H Jr. An Open-Source Privacy-Preserving Large-Scale

Mobile Framework for Cardiovascular Health Monitoring and Intervention Planning

With an Urban African American Population of Young Adults: User-Centered Design

Approach. JMIR Form Res. 2022 Jan 11;6(1):e25444. doi: 10.2196/25444. PMID:

35014970; PMCID: PMC8790689.

245: Fox S, Brown LJE, Antrobus S, Brough D, Drake RJ, Jury F, Leroi I, Parry-

Jones AR, Machin M. Co-design of a Smartphone App for People Living With

Dementia by Applying Agile, Iterative Co-design Principles: Development and

Usability Study. JMIR Mhealth Uhealth. 2022 Jan 14;10(1):e24483. doi:

10.2196/24483. PMID: 35029539; PMCID: PMC8800089.

246: Walker VR, Schmitt CP, Wolfe MS, Nowak AJ, Kulesza K, Williams AR, Shin R,

Cohen J, Burch D, Stout MD, Shipkowski KA, Rooney AA. Evaluation of a semi-

automated data extraction tool for public health literature-based reviews:

Dextr. Environ Int. 2022 Jan 15;159:107025. doi: 10.1016/j.envint.2021.107025.

Epub 2021 Dec 14. PMID: 34920276.

247: Rietze S, Zacher H. Relationships between Agile Work Practices and

Occupational Well-Being: The Role of Job Demands and Resources. Int J Environ

Res Public Health. 2022 Jan 23;19(3):1258. doi: 10.3390/ijerph19031258. PMID:

35162278; PMCID: PMC8835693.

248: Tomlinson E, Goodman J, Loftus M, Bitto S, Carpenter E, Oddo R, Judis L,

Ali S, Robinson WE, Carver M, Ganea M, McDonnell K, O'Neill D, Starbuck J,

Johnson E, Meister E, Pohl J, Spildener J, Shurtleff S, Sovie S, Melendez C,

Krebs P, Riley JD, Wensel C, Astbury C, Azzato EM, Bosler DS, Brock JE, Cook JR,

Cheng YW, Tu ZJ, Cruise M, Henricks WH, Farkas DH. A Model for Design and

Implementation of a Laboratory Information-Management System Specific for

Molecular Pathology Laboratory Operations. J Mol Diagn. 2022 Jan

31:S1525-1578(22)00012-5. doi: 10.1016/j.jmoldx.2022.01.002. Epub ahead of

print. PMID: 35101595.
