# Supplementary material for: Role of Agile in Digital Public Health Transformation
Source: Front Public Health. 2022 May 12;10:899874. doi: 10.3389/fpubh.2022.899874 (PMC9134062; doi:10.3389/fpubh.2022.899874)
Supplement: Supplementary file 2 [file Data_Sheet_2.DOCX]

1: Thunberg KA, Hallberg LR. The need for organizational development in pain

clinics: a case study. Disabil Rehabil. 2002 Sep 20;24(14):755-62. doi:

10.1080/09638280210124356. PMID: 12396661.

2: Lansang MA, Dennis R. Building capacity in health research in the developing

world. Bull World Health Organ. 2004 Oct;82(10):764-70. PMID: 15643798; PMCID:

PMC2623028.

3: Towill DR, Christopher M. An evolutionary approach to the architecture of

effective healthcare delivery systems. J Health Organ Manag. 2005;19(2):130-47.

doi: 10.1108/14777260510600059. PMID: 16119052.

4: Carrico R, Goss L. Syndromic surveillance: hospital emergency department

participation during the Kentucky Derby Festival. Disaster Manag Response. 2005

Jul-Sep;3(3):73-9. doi: 10.1016/j.dmr.2005.04.003. PMID: 15986027; PMCID:

PMC7110958.

5: Kitzmiller R, Hunt E, Sproat SB. Adopting best practices: "Agility" moves

from software development to healthcare project management. Comput Inform Nurs.

2006 Mar-Apr;24(2):75-82; quiz 83-4. doi: 10.1097/00024665-200603000-00005.

PMID: 16554690.

6: Burgess L, Sargent J. Enhancing user acceptance of mandated mobile health

information systems: the ePOC (electronic point-of-care project) experience.

Stud Health Technol Inform. 2007;129(Pt 2):1088-92. PMID: 17911883.

7: Chung J, Pankey E, Norris RJ. Agile informatics: application of agile project

management to the development of a personal health application. AMIA Annu Symp

Proc. 2007 Oct 11:914. PMID: 18694014.

8: Hufnagel SP. National electronic health record interoperability chronology.

Mil Med. 2009 May;174(5 Suppl):35-42. doi: 10.7205/milmed-d-03-9708. PMID:

19562960.

9: Krause P, de Lusignan S. Procuring interoperability at the expense of

usability: a case study of UK National Programme for IT assurance process. Stud

Health Technol Inform. 2010;155:143-9. PMID: 20543322.

10: Johannessen LK, Gammon D. Pilot users in agile development processes:

motivational factors. Stud Health Technol Inform. 2010;157:47-52. PMID:

20543366.

11: Chen YP, Hsieh SH, Cheng PH, Chien TN, Chen HS, Luh JJ, Lai JS, Lai F, Chen

SJ. An agile enterprise regulation architecture for health information security

management. Telemed J E Health. 2010 Sep;16(7):807-17. doi:

10.1089/tmj.2010.0023. PMID: 20815748; PMCID: PMC2956519.

12: Wolf JA. Sustaining high performance: dynamic balancing in an otherwise

unbalanced system. Adv Health Care Manag. 2011;10:367-84. doi:

10.1108/s1474-8231(2011)0000010027. PMID: 21887955.

13: Papakonstantinou D, Poulymenopoulou M, Malamateniou F, Vassilacopoulos G. A

cloud-based semantic wiki for user training in healthcare process management.

Stud Health Technol Inform. 2011;169:93-7. PMID: 21893721.

14: Senathirajah Y, Bakken S. Important ingredients for health adaptive

information systems. Stud Health Technol Inform. 2011;169:280-4. PMID: 21893757.

15: Figueira S, Machado VC, Nunes IL. Integration of human factors principles in

LARG organizations--a conceptual model. Work. 2012;41 Suppl 1:1712-9. doi:

10.3233/WOR-2012-0374-1712. PMID: 22316960.

16: Stefancyk A, Hancock B, Meadows MT. The nurse manager: change agent, change

coach? Nurs Adm Q. 2013 Jan-Mar;37(1):13-7. doi: 10.1097/NAQ.0b013e31827514f4.

PMID: 23222749.

17: Lewis S. Is the deck stacked against fundamental health human resources

redesign in Canada? Healthc Pap. 2013;13(2):23-7; discussion 52-5. doi:

10.12927/hcpap.2013.23522. PMID: 24131811.

18: Treiger TM, Fink-Samnick E. COLLABORATE©: a universal competency-based

paradigm for professional case management, part i: introduction, historical

validation, and competency presentation. Prof Case Manag. 2013 May-

Jun;18(3):122-35; quiz 136-7. doi: 10.1097/NCM.0b013e31828562c0. PMID: 23584522.

19: Treiger TM, Fink-Samnick E. COLLABORATE©: A Universal Competency-Based

Paradigm for Professional Case Management, Part II: Competency Clarification.

Prof Case Manag. 2013 Sep-Oct;18(5):219-43; quiz 244-5. doi:

10.1097/NCM.0b013e31829c8a3a. PMID: 23925073.

20: Dickens PM. Facilitating emergent change in a healthcare setting. Healthc

Manage Forum. 2013 Autumn;26(3):116-26. English, French. doi:

10.1016/j.hcmf.2013.05.003. PMID: 24409579.

21: Lindgren H, Lundin-Olsson L, Pohl P, Sandlund M. End users transforming

experiences into formal information and process models for personalised health

interventions. Stud Health Technol Inform. 2014;205:378-82. PMID: 25160210.

22: Gottfredson C, Stroud C, Jackson M, Stevenson RL, Archer J. Optimizing

learning in healthcare: how Island Health is evolving to learn at the speed of

change. Healthc Q. 2014;17(4):46-51. doi: 10.12927/hcq.2015.24117. PMID:

25906465.

23: Duke JD, Morea J, Mamlin B, Martin DK, Simonaitis L, Takesue BY, Dixon BE,

Dexter PR. Regenstrief Institute's Medical Gopher: a next-generation homegrown

electronic medical record system. Int J Med Inform. 2014 Mar;83(3):170-9. doi:

10.1016/j.ijmedinf.2013.11.004. Epub 2013 Dec 14. PMID: 24373714.

24: Birkinshaw J. Beware the next big thing. Harv Bus Rev. 2014 May;92(5):50-7,

134. PMID: 24956869.

25: Horvath MM, Rusincovitch SA, Brinson S, Shang HC, Evans S, Ferranti JM.

Modular design, application architecture, and usage of a self-service model for

enterprise data delivery: the Duke Enterprise Data Unified Content Explorer

(DEDUCE). J Biomed Inform. 2014 Dec;52:231-42. doi: 10.1016/j.jbi.2014.07.006.

Epub 2014 Jul 19. PMID: 25051403; PMCID: PMC4335712.

26: Tolf S, Nyström ME, Tishelman C, Brommels M, Hansson J. Agile, a guiding

principle for health care improvement? Int J Health Care Qual Assur.

2015;28(5):468-93. doi: 10.1108/IJHCQA-04-2014-0044. PMID: 26020429.

27: Banos O, Villalonga C, Garcia R, Saez A, Damas M, Holgado-Terriza JA, Lee S,

Pomares H, Rojas I. Design, implementation and validation of a novel open

framework for agile development of mobile health applications. Biomed Eng

Online. 2015;14 Suppl 2(Suppl 2):S6. doi: 10.1186/1475-925X-14-S2-S6. Epub 2015

Aug 13. PMID: 26329639; PMCID: PMC4547155.

28: Prakash G. Steering healthcare service delivery: a regulatory perspective.

Int J Health Care Qual Assur. 2015;28(2):173-92. doi:

10.1108/IJHCQA-03-2014-0036. Erratum in: Int J Health Care Qual Assur.

2016;29(1): doi: 10.1108/IJHCQA-02-2016-45. PMID: 26335169.

29: Raghu A, Praveen D, Peiris D, Tarassenko L, Clifford G. Engineering a mobile

health tool for resource-poor settings to assess and manage cardiovascular

disease risk: SMARThealth study. BMC Med Inform Decis Mak. 2015 Apr 29;15:36.

doi: 10.1186/s12911-015-0148-4. PMID: 25924825; PMCID: PMC4430914.

30: Wright A, Sittig DF, Ash JS, Erickson JL, Hickman TT, Paterno M, Gebhardt E,

McMullen C, Tsurikova R, Dixon BE, Fraser G, Simonaitis L, Sonnenberg FA,

Middleton B. Lessons learned from implementing service-oriented clinical

decision support at four sites: A qualitative study. Int J Med Inform. 2015

Nov;84(11):901-11. doi: 10.1016/j.ijmedinf.2015.08.008. Epub 2015 Aug 20. PMID:

26343972.

31: Cormack J, Nath C, Milward D, Raja K, Jonnalagadda SR. Agile text mining for

the 2014 i2b2/UTHealth Cardiac risk factors challenge. J Biomed Inform. 2015

Dec;58 Suppl(0):S120-S127. doi: 10.1016/j.jbi.2015.06.030. Epub 2015 Jul 22.

PMID: 26209007; PMCID: PMC4737484.

32: Lindoerfer D, Mansmann U. Proposing an Evidence-Based Strategy for Software

Requirements Engineering. Stud Health Technol Inform. 2016;228:648-52. PMID:

27577464.

33: Neinstein A, Wong J, Look H, Arbiter B, Quirk K, McCanne S, Sun Y, Blum M,

Adi S. A case study in open source innovation: developing the Tidepool Platform

for interoperability in type 1 diabetes management. J Am Med Inform Assoc. 2016

Mar;23(2):324-32. doi: 10.1093/jamia/ocv104. Epub 2015 Sep 2. PMID: 26338218;

PMCID: PMC4784555.

34: Tweya H, Feldacker C, Gadabu OJ, Ng'ambi W, Mumba SL, Phiri D, Kamvazina L,

Mwakilama S, Kanyerere H, Keiser O, Mwafilaso J, Kamba C, Egger M, Jahn A,

Simwaka B, Phiri S. Developing a point-of-care electronic medical record system

for TB/HIV co-infected patients: experiences from Lighthouse Trust, Lilongwe,

Malawi. BMC Res Notes. 2016 Mar 5;9:146. doi: 10.1186/s13104-016-1943-4. PMID:

26945749; PMCID: PMC4779573.

35: Gale TC, Chatterjee A, Mellor NE, Allan RJ. Health Worker Focused

Distributed Simulation for Improving Capability of Health Systems in Liberia.

Simul Healthc. 2016 Apr;11(2):75-81. doi: 10.1097/SIH.0000000000000156. PMID:

27043091.

36: Sanford JA, Kadry B, Oakes D, Macario A, Schmiesing C. The Heart of the

Matter: Increasing Quality and Charge Capture from Intraoperative

Transesophageal Echocardiography. A A Case Rep. 2016 Apr 15;6(8):249-52. doi:

10.1213/XAA.0000000000000169. PMID: 27082233.

37: Mishuris RG, Yoder J, Wilson D, Mann D. Integrating data from an online

diabetes prevention program into an electronic health record and clinical

workflow, a design phase usability study. BMC Med Inform Decis Mak. 2016 Jul

11;16:88. doi: 10.1186/s12911-016-0328-x. PMID: 27401606; PMCID: PMC4940704.

38: Lennerz JK, McLaughlin HM, Baron JM, Rasmussen D, Sumbada Shin M, Berners-

Lee N, Miller Batten J, Swoboda KJ, Gala MK, Winter HS, Schmahmann JD, Sweetser

DA, Boswell M, Pacula M, Stenzinger A, Le LP, Hynes W, Rehm HL, Klibanski A,

Black-Schaffer SW, Golden JA, Louis DN, Weiss ST, Iafrate AJ. Health Care

Infrastructure for Financially Sustainable Clinical Genomics. J Mol Diagn. 2016

Sep;18(5):697-706. doi: 10.1016/j.jmoldx.2016.04.003. Epub 2016 Jul 25. PMID:

27471182; PMCID: PMC5397703.

39: Gerwitz RJ. Modern strategy for operational excellence: building agile and

adaptive organizations. J Healthc Prot Manage. 2016 Oct;32(2):121-124. PMID:

29638287.

40: Ortiga AM, Lacerda JT, Natal S, Calvo MC. Avaliação do Serviço de

Atendimento Móvel de Urgência em Santa Catarina, Brasil [Evaluation of the

Mobile Emergency Care Service in Santa Catarina State, Brazil]. Cad Saude

Publica. 2016 Dec 15;32(12):e00176714. Portuguese. doi:

10.1590/0102-311X00176714. PMID: 27992039.

41: Abril-Gonzalez M, Portilla FA, Jaramillo-Mejia MC. Standard Health Level

Seven for Odontological Digital Imaging. Telemed J E Health. 2017

Jan;23(1):63-70. doi: 10.1089/tmj.2015.0251. Epub 2016 Jun 1. PMID: 27248059;

PMCID: PMC5240002.

42: Honey M, Procter P. The Shifting Sands of Nursing Informatics Education:

From Content to Connectivity. Stud Health Technol Inform. 2017;232:31-40. PMID:

28106579.

43: Lawrence B. Finding Like-Minded Partners to Span the Continuum of Care.

Front Health Serv Manage. 2017 Fall;34(1):18-30. doi:

10.1097/HAP.0000000000000013. PMID: 28857975.

44: Albornoz MA, Márquez S, Rubin L, Luna D. Design of a Mobile Application for

Transfusion Medicine. Stud Health Technol Inform. 2017;245:994-998. PMID:

29295250.

45: Weiskopf NG, Khan FJ, Woodcock D, Dorr DA, Cigarroa JE, Cohen AM. A Mixed

Methods Task Analysis of the Implementation and Validation of EHR-Based Clinical

Quality Measures. AMIA Annu Symp Proc. 2017 Feb 10;2016:1229-1237. PMID:

28269920; PMCID: PMC5333295.

46: Agha RA, Jafree DJ, Vella-Baldacchino M, Limb C, Kusu-Orkar TE, Millip MC,

Fowler AJ. Surveying opinions of 149 registrants to the Research Registry:

Awareness of and attitudes towards research registration. Int J Surg. 2017

Mar;39:182-187. doi: 10.1016/j.ijsu.2016.12.040. Epub 2017 Jan 5. PMID:

28063975.

47: Kettelhut VV, Vanschooneveld TC, McClay JC, Mercer DF, Fruhling A, Meza JL.

Empirical Study on the Impact of a Tactical Biosurveillance Information

Visualization on Users' Situational Awareness. Mil Med. 2017

Mar;182(S1):322-329. doi: 10.7205/MILMED-D-16-00143. PMID: 28291493.

48: Sarley D, Mahmud M, Idris J, Osunkiyesi M, Dibosa-Osadolor O, Okebukola P,

Wiwa O. Transforming vaccines supply chains in Nigeria. Vaccine. 2017 Apr

19;35(17):2167-2174. doi: 10.1016/j.vaccine.2016.11.068. PMID: 28364926.

49: Suresh M, Patri R. Agility assessment using fuzzy logic approach: a case of

healthcare dispensary. BMC Health Serv Res. 2017 Jun 9;17(1):394. doi:

10.1186/s12913-017-2332-y. PMID: 28599646; PMCID: PMC5466760.

50: Greenhalgh T, A'Court C, Shaw S. Understanding heart failure; explaining

telehealth - a hermeneutic systematic review. BMC Cardiovasc Disord. 2017 Jun

14;17(1):156. doi: 10.1186/s12872-017-0594-2. PMID: 28615004; PMCID: PMC5471857.

51: Kannan V, Fish JS, Mutz JM, Carrington AR, Lai K, Davis LS, Youngblood JE,

Rauschuber MR, Flores KA, Sara EJ, Bhat DG, Willett DL. Rapid Development of

Specialty Population Registries and Quality Measures from Electronic Health

Record Data*. An Agile Framework. Methods Inf Med. 2017 Jun 14;56(99):e74-e83.

doi: 10.3414/ME16-02-0031. PMID: 28930362; PMCID: PMC5608102.

52: Flood D, Douglas K, Goldberg V, Martinez B, Garcia P, Arbour M, Rohloff P. A

quality improvement project using statistical process control methods for type 2

diabetes control in a resource-limited setting. Int J Qual Health Care. 2017 Aug

1;29(4):593-601. doi: 10.1093/intqhc/mzx051. PMID: 28486632.

53: Oza S, Jazayeri D, Teich JM, Ball E, Nankubuge PA, Rwebembera J, Wing K,

Sesay AA, Kanter AS, Ramos GD, Walton D, Cummings R, Checchi F, Fraser HS.

Development and Deployment of the OpenMRS-Ebola Electronic Health Record System

for an Ebola Treatment Center in Sierra Leone. J Med Internet Res. 2017 Aug

21;19(8):e294. doi: 10.2196/jmir.7881. PMID: 28827211; PMCID: PMC5583502.

54: Holch P, Warrington L, Bamforth LCA, Keding A, Ziegler LE, Absolom K, Hector

C, Harley C, Johnson O, Hall G, Morris C, Velikova G. Development of an

integrated electronic platform for patient self-report and management of adverse

events during cancer treatment. Ann Oncol. 2017 Sep 1;28(9):2305-2311. doi:

10.1093/annonc/mdx317. PMID: 28911065; PMCID: PMC5834137.

55: Tepper NK, Krashin JW, Curtis KM, Cox S, Whiteman MK. Update to CDC's U.S.

Medical Eligibility Criteria for Contraceptive Use, 2016: Revised

Recommendations for the Use of Hormonal Contraception Among Women at High Risk

for HIV Infection. MMWR Morb Mortal Wkly Rep. 2017 Sep 22;66(37):990-994. doi:

10.15585/mmwr.mm6637a6. PMID: 28934178; PMCID: PMC5657782.

56: Bam L, McLaren ZM, Coetzee E, von Leipzig KH. Reducing stock-outs of

essential tuberculosis medicines: a system dynamics modelling approach to supply

chain management. Health Policy Plan. 2017 Oct 1;32(8):1127-1134. doi:

10.1093/heapol/czx057. PMID: 28541542.

57: Amalraj A, Varma K, Jacob J, Divya C, Kunnumakkara AB, Stohs SJ, Gopi S. A

Novel Highly Bioavailable Curcumin Formulation Improves Symptoms and Diagnostic

Indicators in Rheumatoid Arthritis Patients: A Randomized, Double-Blind,

Placebo-Controlled, Two-Dose, Three-Arm, and Parallel-Group Study. J Med Food.

2017 Oct;20(10):1022-1030. doi: 10.1089/jmf.2017.3930. Epub 2017 Aug 29. PMID:

28850308.

58: Escosteguy CC, Pereira AGL, Medronho RA. Three decades of hospital

epidemiology and the challenge of integrating Health Surveillance: reflections

from a case study. Cien Saude Colet. 2017 Oct;22(10):3365-3379. Portuguese,

English. doi: 10.1590/1413-812320172210.17562017. PMID: 29069191.

59: Burner E, Lam CN, DeRoss R, Kagawa-Singer M, Menchine M, Arora S. Using

Mobile Health to Improve Social Support for Low-Income Latino Patients with

Diabetes: A Mixed-Methods Analysis of the Feasibility Trial of TExT-MED + FANS.

Diabetes Technol Ther. 2018 Jan;20(1):39-48. doi: 10.1089/dia.2017.0198. Epub

2017 Dec 11. PMID: 29227155; PMCID: PMC5770080.

60: Shimizu HE, Trindade JS, Mesquita MS, Ramos MC. Evaluation of the

Responsiveness Index of the Family Health Strategy in rural areas. Rev Esc

Enferm USP. 2018;52:e03316. English, Portuguese. doi:

10.1590/s1980-220x2017020203316. Epub 2018 Apr 12. PMID: 29668788.

61: Byron SA, Tran NL, Halperin RF, Phillips JJ, Kuhn JG, de Groot JF, Colman H,

Ligon KL, Wen PY, Cloughesy TF, Mellinghoff IK, Butowski NA, Taylor JW, Clarke

JL, Chang SM, Berger MS, Molinaro AM, Maggiora GM, Peng S, Nasser S, Liang WS,

Trent JM, Berens ME, Carpten JD, Craig DW, Prados MD. Prospective Feasibility

Trial for Genomics-Informed Treatment in Recurrent and Progressive Glioblastoma.

Clin Cancer Res. 2018 Jan 15;24(2):295-305. doi: 10.1158/1078-0432.CCR-17-0963.

Epub 2017 Oct 26. PMID: 29074604; PMCID: PMC7516926.

62: Tang T, Lim ME, Mansfield E, McLachlan A, Quan SD. Clinician user

involvement in the real world: Designing an electronic tool to improve

interprofessional communication and collaboration in a hospital setting. Int J

Med Inform. 2018 Feb;110:90-97. doi: 10.1016/j.ijmedinf.2017.11.011. Epub 2017

Nov 22. PMID: 29331258.

63: Lacey Bryant S, Bingham H, Carlyle R, Day A, Ferguson L, Stewart D. Forward

view: advancing health library and knowledge services in England. Health Info

Libr J. 2018 Mar;35(1):70-77. doi: 10.1111/hir.12206. Epub 2018 Jan 10. PMID:

29322613.

64: Soguero-Ruiz C, Mora-Jiménez I, Ramos-López J, Quintanilla Fernández T,

García-García A, Díez-Mazuela D, García-Alberola A, Rojo-Álvarez JL. An

Interoperable System toward Cardiac Risk Stratification from ECG Monitoring. Int

J Environ Res Public Health. 2018 Mar 1;15(3):428. doi: 10.3390/ijerph15030428.

PMID: 29494497; PMCID: PMC5876973.

65: Callahan CM, Bateman DR, Wang S, Boustani MA. State of Science: Bridging the

Science-Practice Gap in Aging, Dementia and Mental Health. J Am Geriatr Soc.

2018 Apr;66 Suppl 1(Suppl 1):S28-S35. doi: 10.1111/jgs.15320. PMID: 29659003;

PMCID: PMC6690193.

66: Tamblyn R, Winslade N, Lee TC, Motulsky A, Meguerditchian A, Bustillo M,

Elsayed S, Buckeridge DL, Couture I, Qian CJ, Moraga T, Huang A. Improving

patient safety and efficiency of medication reconciliation through the

development and adoption of a computer-assisted tool with automated electronic

integration of population-based community drug data: the RightRx project. J Am

Med Inform Assoc. 2018 May 1;25(5):482-495. doi: 10.1093/jamia/ocx107. PMID:

29040609; PMCID: PMC6018649.

67: Ghouila A, Siwo GH, Entfellner JD, Panji S, Button-Simons KA, Davis SZ,

Fadlelmola FM; DREAM of Malaria Hackathon Participants, Ferdig MT, Mulder N.

Hackathons as a means of accelerating scientific discoveries and knowledge

transfer. Genome Res. 2018 May;28(5):759-765. doi: 10.1101/gr.228460.117. Epub

2018 Apr 12. PMID: 29650552; PMCID: PMC5932615.

68: Boustani M, Alder CA, Solid CA. Agile Implementation: A Blueprint for

Implementing Evidence-Based Healthcare Solutions. J Am Geriatr Soc. 2018

Jul;66(7):1372-1376. doi: 10.1111/jgs.15283. Epub 2018 Mar 7. PMID: 29513360.

69: Nazi KM, Turvey CL, Klein DM, Hogan TP. A Decade of Veteran Voices:

Examining Patient Portal Enhancements Through the Lens of User-Centered Design.

J Med Internet Res. 2018 Jul 10;20(7):e10413. doi: 10.2196/10413. PMID:

29991468; PMCID: PMC6058093.

70: Min L, Tian Q, Lu X, Duan H. Modeling EHR with the openEHR approach: an

exploratory study in China. BMC Med Inform Decis Mak. 2018 Aug 29;18(1):75. doi:

10.1186/s12911-018-0650-6. PMID: 30157838; PMCID: PMC6116359.

71: Moore DE Jr, Chappell K, Sherman L, Vinayaga-Pavan M. A conceptual framework

for planning and assessing learning in continuing education activities designed

for clinicians in one profession and/or clinical teams. Med Teach. 2018

Sep;40(9):904-913. doi: 10.1080/0142159X.2018.1483578. Epub 2018 Jul 28. PMID:

30058424.

72: Fisher AM, Mtonga TM, Espino JU, Jonkman LJ, Connor SE, Cappella NK, Douglas

GP. User-centered design and usability testing of RxMAGIC: a prescription

management and general inventory control system for free clinic dispensaries.

BMC Health Serv Res. 2018 Sep 10;18(1):703. doi: 10.1186/s12913-018-3517-8.

PMID: 30200939; PMCID: PMC6131751.

73: Johnson I. Communication Huddles: The Secret of Team Success. J Contin Educ

Nurs. 2018 Oct 1;49(10):451-453. doi: 10.3928/00220124-20180918-04. PMID:

30257027.

74: Rateau Y, Harbouche M, Damaj G, Troussard X. Évaluation de la disparité de

prise en charge des patients avec une leucémie lymphoïde chronique en France

[Evaluation of management disparity in patients with chronic lymphocytic

leukemia in France]. Bull Cancer. 2018 Dec;105(12):1147-1156. French. doi:

10.1016/j.bulcan.2018.08.014. Epub 2018 Nov 2. PMID: 30396506.

75: Ienca M, Vayena E. Dual use in the 21st century: emerging risks and global

governance. Swiss Med Wkly. 2018 Dec 2;148:w14688. doi: 10.4414/smw.2018.14688.

PMID: 30552855.

76: Besiso A, Patrick JD, Dip G, Ho V, Cheng Y. The Impact of an Enterprise

Electronic Medical Record (EEMR) Model vs a Clinical Information System (CIS)

Model on Usability, Efficiency, and Adaptability. AMIA Annu Symp Proc. 2018 Dec

5;2018:242-251. PMID: 30815062; PMCID: PMC6371262.

77: Goodison R, Borycki EM, Kushniruk AW. Use of Agile Project Methodology in

Health Care IT Implementations: A Scoping Review. Stud Health Technol Inform.

2019;257:140-145. PMID: 30741186.

78: Vasa A, Madad S, Larson L, Kraft CS, Vanairsdale S, Grein JD, Garland J,

Butterworth VM, Kratochvil CJ. A Novel Approach to Infectious Disease

Preparedness: Incorporating Investigational Therapeutics and Research Objectives

into Full-Scale Exercises. Health Secur. 2019 Jan/Feb;17(1):54-61. doi:

10.1089/hs.2018.0100. PMID: 30779611.

79: Cahill J, Portales R, McLoughin S, Nagan N, Henrichs B, Wetherall S.

IoT/Sensor-Based Infrastructures Promoting a Sense of Home, Independent Living,

Comfort and Wellness. Sensors (Basel). 2019 Jan 24;19(3):485. doi:

10.3390/s19030485. PMID: 30682864; PMCID: PMC6387202.

80: Lyson HC, Ackerman S, Lyles C, Schillinger D, Williams P, Gourley G, Gupta

R, Handley M, Sarkar U. Redesigning primary care in the safety net: A

qualitative analysis of team-based care implementation. Healthc (Amst). 2019

Mar;7(1):22-29. doi: 10.1016/j.hjdsi.2018.09.004. Epub 2018 Dec 11. PMID:

30552044.

81: Moon LA, Clancy G, Welton J, Harper E. Nursing Value User Stories: A Value

Measurement Method for Linking Nurse Contribution to Patient Outcomes. Comput

Inform Nurs. 2019 Mar;37(3):161-170. doi: 10.1097/CIN.0000000000000520. PMID:

30762611.

82: Sieja A, Markley K, Pell J, Gonzalez C, Redig B, Kneeland P, Lin CT.

Optimization Sprints: Improving Clinician Satisfaction and Teamwork by Rapidly

Reducing Electronic Health Record Burden. Mayo Clin Proc. 2019

May;94(5):793-802. doi: 10.1016/j.mayocp.2018.08.036. Epub 2019 Feb 26. PMID:

30824281.

83: Neumann K, Reichl V, Rong O. Dringender Handlungsbedarf für die Zukunft des

digitalen Krankenhauses [Urgent need of action for the future of digital

hospitals]. HNO. 2019 May;67(5):350-355. German. doi: 10.1007/s00106-019-0655-1.

PMID: 30963220.

84: Jamieson T, Mamdani MM, Etchells E. Linking Quality Improvement and Health

Information Technology through the QI-HIT Figure 8. Appl Clin Inform. 2019

May;10(3):528-533. doi: 10.1055/s-0039-1693456. Epub 2019 Jul 24. PMID:

31340398; PMCID: PMC6656570.

85: Oza S, Wing K, Sesay AA, Boufkhed S, Houlihan C, Vandi L, Sebba SC, McGowan

CR, Cummings R, Checchi F. Improving health information systems during an

emergency: lessons and recommendations from an Ebola treatment centre in Sierra

Leone. BMC Med Inform Decis Mak. 2019 May 27;19(1):100. doi:

10.1186/s12911-019-0817-9. PMID: 31133075; PMCID: PMC6537453.

86: Bricknell M, Finn A, Palmer J. For debate: health service support planning

for large-scale defensive land operations (part 2). J R Army Med Corps. 2019

Jun;165(3):176-179. doi: 10.1136/jramc-2018-000994. Epub 2018 Aug 19. PMID:

30127067.

87: Chen BA, Blithe DL, Muraguri GR, Lance AA, Carr BR, Jensen JT, Kimble TD,

Murthy AS, Schreiber CA, Thomas MA, Walsh TL, Westhoff C, Burke AE.

Acceptability of the Woman's Condom in a phase III multicenter open-label study.

Contraception. 2019 Jun;99(6):357-362. doi: 10.1016/j.contraception.2019.02.006.

Epub 2019 Mar 6. PMID: 30849305; PMCID: PMC7363037.

88: Preisz A. Fast and slow thinking; and the problem of conflating clinical

reasoning and ethical deliberation in acute decision-making. J Paediatr Child

Health. 2019 Jun;55(6):621-624. doi: 10.1111/jpc.14447. Epub 2019 Apr 1. PMID:

30932284.

89: Clark KD, Woodson TT, Holden RJ, Gunn R, Cohen DJ. Translating Research into

Agile Development (TRIAD): Development of Electronic Health Record Tools for

Primary Care Settings. Methods Inf Med. 2019 Jun;58(1):1-8. doi:

10.1055/s-0039-1692464. Epub 2019 Jul 5. PMID: 31277082; PMCID: PMC6823924.

90: Allsop MJ, Johnson O, Taylor S, Hackett J, Allen P, Bennett MI, Bewick BM.

Multidisciplinary Software Design for the Routine Monitoring and Assessment of

Pain in Palliative Care Services: The Development of PainCheck. JCO Clin Cancer

Inform. 2019 Sep;3:1-17. doi: 10.1200/CCI.18.00120. PMID: 31577449; PMCID:

PMC6873922.

91: Kannan V, Basit MA, Bajaj P, Carrington AR, Donahue IB, Flahaven EL, Medford

R, Melaku T, Moran BA, Saldana LE, Willett DL, Youngblood JE, Toomay SM. User

stories as lightweight requirements for agile clinical decision support

development. J Am Med Inform Assoc. 2019 Nov 1;26(11):1344-1354. doi:

10.1093/jamia/ocz123. PMID: 31512730; PMCID: PMC6798563.

92: Pham Q, Shaw J, Morita PP, Seto E, Stinson JN, Cafazzo JA. The Service of

Research Analytics to Optimize Digital Health Evidence Generation: Multilevel

Case Study. J Med Internet Res. 2019 Nov 11;21(11):e14849. doi: 10.2196/14849.

PMID: 31710296; PMCID: PMC6878108.

93: Borrelli B, Henshaw M, Endrighi R, Adams WG, Heeren T, Rosen RK, Bock B,

Werntz S. An Interactive Parent-Targeted Text Messaging Intervention to Improve

Oral Health in Children Attending Urban Pediatric Clinics: Feasibility

Randomized Controlled Trial. JMIR Mhealth Uhealth. 2019 Nov 11;7(11):e14247.

doi: 10.2196/14247. PMID: 31710306; PMCID: PMC6878100.

94: Hales AA, Cable D, Crossley E, Findlay C, Rew DA. Design and implementation

of the stacked, synchronised and iconographic timeline-structured electronic

patient record in a UK NHS Global Digital Exemplar hospital. BMJ Health Care

Inform. 2019 Dec;26(1):e100025. doi: 10.1136/bmjhci-2019-100025. PMID: 31874854;

PMCID: PMC7252964.

95: Aaronson EL, White BA, Black L, Sonis JD, Mort EA. Using Design Thinking to

Improve Patient-Provider Communication in the Emergency Department. Qual Manag

Health Care. 2020 Jan/Mar;29(1):30-34. doi: 10.1097/QMH.0000000000000239. PMID:

31855933.

96: Banerjee A, Sculean A, Petersen PE. 'MI' reflections on a pandemic-governed

2020. Oral Health Prev Dent. 2020;18(1):1-2. doi: 10.3290/j.ohpd.b871931. PMID:

33499553.

97: Blake H, Somerset S, Evans C. Development and Fidelity Testing of the

Test@Work Digital Toolkit for Employers on Workplace Health Checks and Opt-In

HIV Testing. Int J Environ Res Public Health. 2020 Jan 6;17(1):379. doi:

10.3390/ijerph17010379. PMID: 31935985; PMCID: PMC6982120.

98: Lynch M, Kodate N. Professional practice following regulatory change: An

evaluation using principles of "Better Regulation". Res Social Adm Pharm. 2020

Feb;16(2):208-215. doi: 10.1016/j.sapharm.2019.05.007. Epub 2019 May 18. PMID:

31133538.

99: Paul M, Jena LK, Sahoo K. Workplace Spirituality and Workforce Agility: A

Psychological Exploration Among Teaching Professionals. J Relig Health. 2020

Feb;59(1):135-153. doi: 10.1007/s10943-019-00918-3. PMID: 31549287.

100: López M, Jiménez JM, Martín-Gil B, Fernández-Castro M, Cao MJ, Frutos M,

Castro MJ. The impact of an educational intervention on nursing students'

critical thinking skills: A quasi-experimental study. Nurse Educ Today. 2020

Feb;85:104305. doi: 10.1016/j.nedt.2019.104305. Epub 2019 Nov 21. PMID:

31778861.

101: Ghosh R, Kempf D, Pufko A, Barrios Martinez LF, Davis CM, Sethi S.

Automation Opportunities in Pharmacovigilance: An Industry Survey. Pharmaceut

Med. 2020 Feb;34(1):7-18. doi: 10.1007/s40290-019-00320-0. PMID: 32036574.

102: Improta G, Guizzi G, Ricciardi C, Giordano V, Ponsiglione AM, Converso G,

Triassi M. Agile Six Sigma in Healthcare: Case Study at Santobono Pediatric

Hospital. Int J Environ Res Public Health. 2020 Feb 7;17(3):1052. doi:

10.3390/ijerph17031052. PMID: 32046052; PMCID: PMC7037742.

103: Kaczor C, Cole JW, Sapko MM, Kappeler KH. Realizing the vision for

pediatric pharmacy practice advancement through strategic planning and

implementation. Am J Health Syst Pharm. 2020 Mar 5;77(6):466-473. doi:

10.1093/ajhp/zxz340. PMID: 31960893.

104: López Seguí F, Franch Parella J, Gironès García X, Mendioroz Peña J, García

Cuyàs F, Adroher Mas C, García-Altés A, Vidal-Alaball J. A Cost-Minimization

Analysis of a Medical Record-based, Store and Forward and Provider-to-provider

Telemedicine Compared to Usual Care in Catalonia: More Agile and Efficient,

Especially for Users. Int J Environ Res Public Health. 2020 Mar 18;17(6):2008.

doi: 10.3390/ijerph17062008. PMID: 32197434; PMCID: PMC7143363.

105: Burningham Z, Jackson GL, Kelleher J, Stevens M, Morris I, Cohen J, Maloney

G, Vaughan CP. The Enhancing Quality of Prescribing Practices for Older Veterans

Discharged From the Emergency Department (EQUIPPED) Potentially Inappropriate

Medication Dashboard: A Suitable Alternative to the In-person Academic Detailing

and Standardized Feedback Reports of Traditional EQUIPPED? Clin Ther. 2020

Apr;42(4):573-582. doi: 10.1016/j.clinthera.2020.02.013. Epub 2020 Mar 25. PMID:

32222360.

106: Blake H, Bermingham F, Johnson G, Tabner A. Mitigating the Psychological

Impact of COVID-19 on Healthcare Workers: A Digital Learning Package. Int J

Environ Res Public Health. 2020 Apr 26;17(9):2997. doi: 10.3390/ijerph17092997.

PMID: 32357424; PMCID: PMC7246821.

107: Tavernier SS, Beck SL. Design and Evaluation of the Electronic Patient-

Generated Index. Nurs Res. 2020 May/Jun;69(3):227-232. doi:

10.1097/NNR.0000000000000405. PMID: 31688339.

108: Carey VJ, Ramos M, Stubbs BJ, Gopaulakrishnan S, Oh S, Turaga N, Waldron L,

Morgan M. Global Alliance for Genomics and Health Meets Bioconductor: Toward

Reproducible and Agile Cancer Genomics at Cloud Scale. JCO Clin Cancer Inform.

2020 May;4:472-479. doi: 10.1200/CCI.19.00111. PMID: 32453635; PMCID:

PMC7265787.

109: Noble L, Scott L, Stewart-Isherwood L, Molifi SJ, Sanne I, Da Silva P,

Stevens W. Continuous quality monitoring in the field: an evaluation of the

performance of the Fio Deki Reader™ for rapid HIV testing in South Africa. BMC

Infect Dis. 2020 May 4;20(1):320. doi: 10.1186/s12879-020-4932-0. PMID:

32366227; PMCID: PMC7199324.

110: Schulz WL, Durant TJS, Torre CJ Jr, Hsiao AL, Krumholz HM. Agile Health

Care Analytics: Enabling Real-Time Disease Surveillance With a Computational

Health Platform. J Med Internet Res. 2020 May 28;22(5):e18707. doi:

10.2196/18707. PMID: 32442130; PMCID: PMC7257473.

111: Schinköthe T, Gabri MR, Mitterer M, Gouveia P, Heinemann V, Harbeck N,

Subklewe M. A Web- and App-Based Connected Care Solution for COVID-19 In- and

Outpatient Care: Qualitative Study and Application Development. JMIR Public

Health Surveill. 2020 Jun 1;6(2):e19033. doi: 10.2196/19033. PMID: 32406855;

PMCID: PMC7265653.

112: Schleimer E, Pearce J, Barnecut A, Rowles W, Lizee A, Klein A, Block VJ,

Santaniello A, Renschen A, Gomez R, Keshavan A, Gelfand JM, Henry RG, Hauser SL,

Bove R. A Precision Medicine Tool for Patients With Multiple Sclerosis (the Open

MS BioScreen): Human-Centered Design and Development. J Med Internet Res. 2020

Jul 6;22(7):e15605. doi: 10.2196/15605. PMID: 32628124; PMCID: PMC7381029.

113: Katz JN, Sinha SS, Alviar CL, Dudzinski DM, Gage A, Brusca SB, Flanagan MC,

Welch T, Geller BJ, Miller PE, Leonardi S, Bohula EA, Price S, Chaudhry SP,

Metkus TS, O'Brien CG, Sionis A, Barnett CF, Jentzer JC, Solomon MA, Morrow DA,

van Diepen S. COVID-19 and Disruptive Modifications to Cardiac Critical Care

Delivery: JACC Review Topic of the Week. J Am Coll Cardiol. 2020 Jul

7;76(1):72-84. doi: 10.1016/j.jacc.2020.04.029. Epub 2020 Apr 16. PMID:

32305402; PMCID: PMC7161519.

114: Weemaes M, Martens S, Cuypers L, Van Elslande J, Hoet K, Welkenhuysen J,

Goossens R, Wouters S, Houben E, Jeuris K, Laenen L, Bruyninckx K, Beuselinck K,

André E, Depypere M, Desmet S, Lagrou K, Van Ranst M, Verdonck AKLC, Goveia J.

Laboratory information system requirements to manage the COVID-19 pandemic: A

report from the Belgian national reference testing center. J Am Med Inform

Assoc. 2020 Aug 1;27(8):1293-1299. doi: 10.1093/jamia/ocaa081. PMID: 32348469;

PMCID: PMC7197526.

115: O'Reilly GM, Mitchell RD, Rajiv P, Wu J, Brennecke H, Brichko L, Noonan MP,

Hiller R, Mitra B, Luckhoff C, Paton A, Smit V, Santamaria MJ, Cameron PA.

Epidemiology and clinical features of emergency department patients with

suspected COVID-19: Initial results from the COVID-19 Emergency Department

Quality Improvement Project (COVED-1). Emerg Med Australas. 2020

Aug;32(4):638-645. doi: 10.1111/1742-6723.13540. Epub 2020 May 18. PMID:

32378797.

116: Elhadi M, Msherghi A, Alkeelani M, Zorgani A, Zaid A, Alsuyihili A, Buzreg

A, Ahmed H, Elhadi A, Khaled A, Boughididah T, Khel S, Abdelkabir M, Gaffaz R,

Bahroun S, Alhashimi A, Biala M, Abulmida S, Elharb A, Abukhashem M, Elgzairi M,

Alghanai E, Khaled T, Boushi E, Ben Saleim N, Mughrabi H, Alnafati N, Alwarfalli

M, Elmabrouk A, Alhaddad S, Madi F, Madi M, Elkhfeefi F, Ismaeil M, Faraag B,

Badi M, Al-Agile A, Eisay M, Ahmid J, Elmabrouk O, Bin Alshiteewi F, Alameen H,

Bikhayr H, Aleiyan T, Almiqlash B, Subhi M, Fadel M, Yahya H, Alkot S, Alhadi A,

Abdullah A, Atewa A, Amshai A. Assessment of Healthcare Workers' Levels of

Preparedness and Awareness Regarding COVID-19 Infection in Low-Resource

Settings. Am J Trop Med Hyg. 2020 Aug;103(2):828-833. doi:

10.4269/ajtmh.20-0330. Epub 2020 Jun 18. PMID: 32563273; PMCID: PMC7410457.

117: Tobias G, Spanier AB. Developing a Mobile App (iGAM) to Promote Gingival

Health by Professional Monitoring of Dental Selfies: User-Centered Design

Approach. JMIR Mhealth Uhealth. 2020 Aug 14;8(8):e19433. doi: 10.2196/19433.

PMID: 32795985; PMCID: PMC7455872.

118: De Biase S, Cook L, Skelton DA, Witham M, Ten Hove R. The COVID-19

rehabilitation pandemic. Age Ageing. 2020 Aug 24;49(5):696-700. doi:

10.1093/ageing/afaa118. PMID: 32470131; PMCID: PMC7314277.

119: Faust O, Lei N, Chew E, Ciaccio EJ, Acharya UR. A Smart Service Platform

for Cost Efficient Cardiac Health Monitoring. Int J Environ Res Public Health.

2020 Aug 30;17(17):6313. doi: 10.3390/ijerph17176313. PMID: 32872667; PMCID:

PMC7504315.

120: Olsen O, Greene A, Makrides T, Delport A. Large-Scale Air Medical

Operations in the Age of Coronavirus Disease 2019: Early Leadership Lessons From

the Front Lines of British Columbia. Air Med J. 2020 Sep-Oct;39(5):340-342. doi:

10.1016/j.amj.2020.04.015. Epub 2020 May 7. PMID: 33012469; PMCID: PMC7203048.

121: Means AR, Wagner AD, Kern E, Newman LP, Weiner BJ. Implementation Science

to Respond to the COVID-19 Pandemic. Front Public Health. 2020 Sep 2;8:462. doi:

10.3389/fpubh.2020.00462. PMID: 32984248; PMCID: PMC7493639.

122: Elmore JG, Wang PC, Kerr KF, Schriger DL, Morrison DE, Brookmeyer R,

Pfeffer MA, Payne TH, Currier JS. Excess Patient Visits for Cough and Pulmonary

Disease at a Large US Health System in the Months Prior to the COVID-19

Pandemic: Time-Series Analysis. J Med Internet Res. 2020 Sep 10;22(9):e21562.

doi: 10.2196/21562. PMID: 32791492; PMCID: PMC7485935.

123: Franceschi D, Suarez MM, Ruiz JW, Seo D, Merchant NB. A Novel

Interdisciplinary Iterative Approach for Optimizing the Electronic Health Record

to Improve Perioperative Efficiency. Ann Surg. 2020 Oct;272(4):669-675. doi:

10.1097/SLA.0000000000004347. PMID: 32932324.

124: Koehlmoos TP, Banaag A, Madsen CK, Adirim T. Child Health As A National

Security Issue: Obesity And Behavioral Health Conditions Among Military

Children. Health Aff (Millwood). 2020 Oct;39(10):1719-1727. doi:

10.1377/hlthaff.2020.00712. PMID: 33017245.

125: Martin N. Finding a New Normal: Hospital Governance Best Practices during

COVID-19. Healthc Q. 2020 Oct;23(3):24-28. doi: 10.12927/hcq.2020.26337. PMID:

33243362.

126: Narla NP, Surmeli A, Kivlehan SM. Agile Application of Digital Health

Interventions during the COVID-19 Refugee Response. Ann Glob Health. 2020 Oct

15;86(1):135. doi: 10.5334/aogh.2995. PMID: 33117656; PMCID: PMC7566526.

127: Sasangohar F, Moats J, Mehta R, Peres SC. Disaster Ergonomics: Human

Factors in COVID-19 Pandemic Emergency Management. Hum Factors. 2020

Nov;62(7):1061-1068. doi: 10.1177/0018720820939428. Epub 2020 Jul 10. PMID:

32648781.

128: Veepanattu P, Singh S, Mendelson M, Nampoothiri V, Edathadatil F, Surendran

S, Bonaconsa C, Mbamalu O, Ahuja S, Birgand G, Tarrant C, Sevdalis N, Ahmad R,

Castro-Sanchez E, Holmes A, Charani E. Building resilient and responsive

research collaborations to tackle antimicrobial resistance-Lessons learnt from

India, South Africa, and UK. Int J Infect Dis. 2020 Nov;100:278-282. doi:

10.1016/j.ijid.2020.08.057. Epub 2020 Aug 27. PMID: 32860949; PMCID: PMC7449941.

129: Mehta J, Yates T, Smith P, Henderson D, Winteringham G, Burns A. Rapid

implementation of Microsoft Teams in response to COVID-19: one acute healthcare

organisation's experience. BMJ Health Care Inform. 2020 Nov;27(3):e100209. doi:

10.1136/bmjhci-2020-100209. PMID: 33177050; PMCID: PMC7661347.

130: Abel KM, Bee P, Gega L, Gellatly J, Kolade A, Hunter D, Callender C, Carter

LA, Meacock R, Bower P, Stanley N, Calam R, Wolpert M, Stewart P, Emsley R, Holt

K, Linklater H, Douglas S, Stokes-Crossley B, Green J. An intervention to

improve the quality of life in children of parents with serious mental illness:

the Young SMILES feasibility RCT. Health Technol Assess. 2020 Nov;24(59):1-136.

doi: 10.3310/hta24590. PMID: 33196410; PMCID: PMC7701992.

131: Franco Miguel JL, Fullana Belda C, Cordero Ferrera JM, Polo C, Nuño-Solinís

R. Efficiency in chronic illness care coordination: public-private collaboration

models vs. traditional management. BMC Health Serv Res. 2020 Nov 16;20(1):1044.

doi: 10.1186/s12913-020-05894-z. PMID: 33198716; PMCID: PMC7667775.

132: Wang S, Hanneman P, Xu C, Gao S, Allen D, Golovyan D, Kheir YN, Fowler N,

Austrom M, Khan S, Boustani M, Khan B. Critical Care Recovery Center: a model of

agile implementation in intensive care unit (ICU) survivors. Int Psychogeriatr.

2020 Dec;32(12):1409-1418. doi: 10.1017/S1041610219000553. Epub 2019 Aug 30.

PMID: 31466536; PMCID: PMC7048643.

133: Murphy BP, O'Raghallaigh P, Carr M. Nurturing the digital baby: Open

innovation for development and optimization. Health Informatics J. 2020

Dec;26(4):2407-2421. doi: 10.1177/1460458220906067. Epub 2020 Feb 25. PMID:

32098558.

134: Miles J, Jones C. The Light Role CCP: A Blueprint for Far Forward Medical

Support to Contemporary Operations. BMJ Mil Health. 2020 Dec;166(6):433-438.

doi: 10.1136/bmjmilitary-2020-001435. Epub 2020 May 17. PMID: 32423897.

135: Xue CL, Shu YS, Hayter M, Lee A. Experiences of nurses involved in natural

disaster relief: A meta-synthesis of qualitative literature. J Clin Nurs. 2020

Dec;29(23-24):4514-4531. doi: 10.1111/jocn.15476. Epub 2020 Sep 17. PMID:

32869888; PMCID: PMC7756389.

136: DeKeyser GJ, Brodke DS, Saltzman CL, Lawrence BD. Response to the

Coronavirus Disease 2019 Pandemic by the Spine Division at a Level-I Academic

Referral Center. J Am Acad Orthop Surg. 2020 Dec 15;28(24):1003-1008. doi:

10.5435/JAAOS-D-20-00493. PMID: 33278260.

137: Leppla L, Hobelsberger S, Rockstein D, Werlitz V, Pschenitza S, Heidegger

P, De Geest S, Valenta S, Teynor A; SMILe study team. Implementation Science

Meets Software Development to Create eHealth Components for an Integrated Care

Model for Allogeneic Stem Cell Transplantation Facilitated by eHealth: The SMILe

Study as an Example. J Nurs Scholarsh. 2021 Jan;53(1):35-45. doi:

10.1111/jnu.12621. Epub 2020 Dec 21. PMID: 33348461.

138: Bolislis WR, de Lucia ML, Dolz F, Mo R, Nagaoka M, Rodriguez H, Woon ML, Yu

W, Kühler TC. Regulatory Agilities in the Time of COVID-19: Overview, Trends,

and Opportunities. Clin Ther. 2021 Jan;43(1):124-139. doi:

10.1016/j.clinthera.2020.11.015. Epub 2020 Nov 30. PMID: 33353762; PMCID:

PMC7703519.

139: Bone EA, Tochkin J. The benefits of lessons learned: The COVID-19

experience in the Canadian province of Alberta. J Bus Contin Emer Plan. 2021 Jan

1;15(2):140-150. PMID: 35016748.

140: Mao Z, Yao H, Zou Q, Zhang W, Dong Y. Digital Contact Tracing Based on a

Graph Database Algorithm for Emergency Management During the COVID-19 Epidemic:

Case Study. JMIR Mhealth Uhealth. 2021 Jan 22;9(1):e26836. doi: 10.2196/26836.

PMID: 33460389; PMCID: PMC7837510.

141: Pach D, Rogge AA, Wang J, Witt CM. Five Lessons Learned From Randomized

Controlled Trials on Mobile Health Interventions: Consensus Procedure on

Practical Recommendations for Sustainable Research. JMIR Mhealth Uhealth. 2021

Feb 8;9(2):e20630. doi: 10.2196/20630. PMID: 33555263; PMCID: PMC7899803.

142: Rubiano L, Alexander NDE, Castillo RM, Martínez ÁJ, García Luna JA, Arango

JD, Vargas L, Madriñán P, Hurtado LR, Orobio Y, Rojas CA, Del Corral H, Navarro

A, Gore Saravia N, Aronoff-Spencer E. Adaptation and performance of a mobile

application for early detection of cutaneous leishmaniasis. PLoS Negl Trop Dis.

2021 Feb 11;15(2):e0008989. doi: 10.1371/journal.pntd.0008989. PMID: 33571192;

PMCID: PMC7904137.

143: Tao S, Lhatoo S, Hampson J, Cui L, Zhang GQ. A Bespoke Electronic Health

Record for Epilepsy Care (EpiToMe): Development and Qualitative Evaluation. J

Med Internet Res. 2021 Feb 12;23(2):e22939. doi: 10.2196/22939. PMID: 33576745;

PMCID: PMC7910122.

144: Jeyakumar T, McClure S, Lowe M, Hodges B, Fur K, Javier-Brozo M, Tassone M,

Anderson M, Tripp T, Wiljer D. An Education Framework for Effective

Implementation of a Health Information System: Scoping Review. J Med Internet

Res. 2021 Feb 24;23(2):e24691. doi: 10.2196/24691. PMID: 33625370; PMCID:

PMC7946593.

145: Bains J, Greenwald PW, Mulcare MR, Leyden D, Kim J, Shemesh AJ, Bodnar D,

Farmer B, Steel P, Tanouye R, Kim JW, Lame M, Sharma R. Utilizing Telemedicine

in a Novel Approach to COVID-19 Management and Patient Experience in the

Emergency Department. Telemed J E Health. 2021 Mar;27(3):254-260. doi:

10.1089/tmj.2020.0162. Epub 2020 Aug 18. PMID: 32821027.

146: Brunet F, Malas K, Fleury D. A model of an agile organization designed to

better manage the COVID-19 crisis. Healthc Manage Forum. 2021 Mar;34(2):115-118.

doi: 10.1177/0840470420980478. Epub 2020 Dec 23. PMID: 33353424; PMCID:

PMC7758620.

147: Luckett T, Donkor A, Phillips J, Currow DC, Parker D, Lobb E, Agar MR.

Australian specialist palliative care's response to COVID-19: an anonymous

online survey of service providers. Ann Palliat Med. 2021 Mar;10(3):2747-2757.

doi: 10.21037/apm-20-1760. Epub 2021 Jan 14. PMID: 33474953.

148: Arabi YM, Azoulay E, Al-Dorzi HM, Phua J, Salluh J, Binnie A, Hodgson C,

Angus DC, Cecconi M, Du B, Fowler R, Gomersall CD, Horby P, Juffermans NP,

Kesecioglu J, Kleinpell RM, Machado FR, Martin GS, Meyfroidt G, Rhodes A, Rowan

K, Timsit JF, Vincent JL, Citerio G. How the COVID-19 pandemic will change the

future of critical care. Intensive Care Med. 2021 Mar;47(3):282-291. doi:

10.1007/s00134-021-06352-y. Epub 2021 Feb 22. PMID: 33616696; PMCID: PMC7898492.

149: Sullivan C, Wong I, Adams E, Fahim M, Fraser J, Ranatunga G, Busato M,

McNeil K. Moving Faster than the COVID-19 Pandemic: The Rapid, Digital

Transformation of a Public Health System. Appl Clin Inform. 2021

Mar;12(2):229-236. doi: 10.1055/s-0041-1725186. Epub 2021 Mar 24. PMID:

33763847; PMCID: PMC7990571.

150: Dubuc N, Brière S, Corbin C, N'Bouke A, Bonin L, Delli-Colli N.

Computerized Care-Pathways (CCPs) System to Support Person-Centered, Integrated,

and Proactive Care in Home-Care Settings. Inform Health Soc Care. 2021 Mar

2;46(1):100-111. doi: 10.1080/17538157.2020.1865969. Epub 2021 Jan 6. PMID:

33406972.

151: Hill JR, Harrington AB, Adeoye P, Campbell NL, Holden RJ. Going Remote-

Demonstration and Evaluation of Remote Technology Delivery and Usability

Assessment With Older Adults: Survey Study. JMIR Mhealth Uhealth. 2021 Mar

4;9(3):e26702. doi: 10.2196/26702. PMID: 33606655; PMCID: PMC7935399.

152: Gárate FJ, Chausa P, Whetham J, Jones CI, García F, Cáceres C, Sánchez-

González P, Wallitt E, Gómez EJ, On Behalf Of The EmERGE Consortium. EmERGE

mHealth Platform: Implementation and Technical Evaluation of a Digital Supported

Pathway of Care for Medically Stable HIV. Int J Environ Res Public Health. 2021

Mar 18;18(6):3156. doi: 10.3390/ijerph18063156. PMID: 33803821; PMCID:

PMC8003226.

153: Hannan RJ, Lundholm MK, Brierton D, Chapman NRM. Responding to unforeseen

disasters in a large health system. Am J Health Syst Pharm. 2021 Mar

31;78(8):726-731. doi: 10.1093/ajhp/zxaa358. PMID: 33119088; PMCID: PMC7665326.

154: Phillips JF, MacLeod BB, Kachur SP. Bugs in the Bed: Addressing the

Contradictions of Embedded Science with Agile Implementation Research. Glob

Health Sci Pract. 2021 Mar 31;9(1):55-77. doi: 10.9745/GHSP-D-20-00169. PMID:

33795362; PMCID: PMC8087429.

155: Berlin A, Lovas M, Truong T, Melwani S, Liu J, Liu ZA, Badzynski A,

Carpenter MB, Virtanen C, Morley L, Bhattacharyya O, Escaf M, Moody L, Goldfarb

A, Brzozowski L, Cafazzo J, Chua MLK, Stewart AK, Krzyzanowska MK.

Implementation and Outcomes of Virtual Care Across a Tertiary Cancer Center

During COVID-19. JAMA Oncol. 2021 Apr 1;7(4):597-602. doi:

10.1001/jamaoncol.2020.6982. PMID: 33410867; PMCID: PMC7791400.

156: INSPIRATION Investigators, Sadeghipour P, Talasaz AH, Rashidi F, Sharif-

Kashani B, Beigmohammadi MT, Farrokhpour M, Sezavar SH, Payandemehr P, Dabbagh

A, Moghadam KG, Jamalkhani S, Khalili H, Yadollahzadeh M, Riahi T, Rezaeifar P,

Tahamtan O, Matin S, Abedini A, Lookzadeh S, Rahmani H, Zoghi E, Mohammadi K,

Sadeghipour P, Abri H, Tabrizi S, Mousavian SM, Shahmirzaei S, Bakhshandeh H,

Amin A, Rafiee F, Baghizadeh E, Mohebbi B, Parhizgar SE, Aliannejad R, Eslami V,

Kashefizadeh A, Kakavand H, Hosseini SH, Shafaghi S, Ghazi SF, Najafi A, Jimenez

D, Gupta A, Madhavan MV, Sethi SS, Parikh SA, Monreal M, Hadavand N, Hajighasemi

A, Maleki M, Sadeghian S, Piazza G, Kirtane AJ, Van Tassell BW, Dobesh PP, Stone

GW, Lip GYH, Krumholz HM, Goldhaber SZ, Bikdeli B. Effect of Intermediate-Dose

vs Standard-Dose Prophylactic Anticoagulation on Thrombotic Events,

Extracorporeal Membrane Oxygenation Treatment, or Mortality Among Patients With

COVID-19 Admitted to the Intensive Care Unit: The INSPIRATION Randomized

Clinical Trial. JAMA. 2021 Apr 27;325(16):1620-1630. doi:

10.1001/jama.2021.4152. PMID: 33734299; PMCID: PMC7974835.

157: Lavoie ME, Tay KY, Good G, Buchhalter L, Abbadessa MK, Gaines S, Myers S.

Simulation as a Dynamic Tool to Reorganize Pediatric Emergency Department

Resuscitation During the Coronavirus Disease 2019 Pandemic and Beyond. Pediatr

Emerg Care. 2021 May 1;37(5):286-289. doi: 10.1097/PEC.0000000000002384. PMID:

33903290.

158: Russ MJ, Parish SJ, Mendelowitz R, Mendoza S, Arkow SD, Radosta M, Espinosa

L, Sombrotto LB, Anthony D, Wyman DA, Baptista-Neto L, Wilner PJ. The Interface

of COVID-19 and Inpatient Psychiatry: Our Experience and Lessons Learned. J

Psychiatr Pract. 2021 May 5;27(3):172-183. doi: 10.1097/PRA.0000000000000551.

PMID: 33939371; PMCID: PMC8143149.

159: Lo WC, Wang FC, Lin LY, Jyan HW, Wu HC, Huang YL, Parng IM, Chiou HY.

Enhancing Data Linkage to Break the Chain of COVID-19 Spread: The Taiwan

Experience. J Med Internet Res. 2021 May 7;23(5):e24294. doi: 10.2196/24294.

PMID: 33882019; PMCID: PMC8108927.

160: Beyer J, Collette L, Sauvé N, Daugaard G, Feldman DR, Tandstad T, Tryakin

A, Stahl O, Gonzalez-Billalabeitia E, De Giorgi U, Culine S, de Wit R, Hansen

AR, Bebek M, Terbuch A, Albany C, Hentrich M, Gietema JA, Negaard H, Huddart RA,

Lorch A, Cafferty FH, Heng DYC, Sweeney CJ, Winquist E, Chovanec M, Fankhauser

C, Stark D, Grimison P, Necchi A, Tran B, Heidenreich A, Shamash J, Sternberg

CN, Vaughn DJ, Duran I, Bokemeyer C, Patrikidou A, Cathomas R, Assele S,

Gillessen S; International Germ Cell Cancer Classification Update Consortium.

Survival and New Prognosticators in Metastatic Seminoma: Results From the

IGCCCG-Update Consortium. J Clin Oncol. 2021 May 10;39(14):1553-1562. doi:

10.1200/JCO.20.03292. Epub 2021 Mar 17. PMID: 33729863; PMCID: PMC8099394.

161: Gillessen S, Sauvé N, Collette L, Daugaard G, de Wit R, Albany C, Tryakin

A, Fizazi K, Stahl O, Gietema JA, De Giorgi U, Cafferty FH, Hansen AR, Tandstad

T, Huddart RA, Necchi A, Sweeney CJ, Garcia-Del-Muro X, Heng DYC, Lorch A,

Chovanec M, Winquist E, Grimison P, Feldman DR, Terbuch A, Hentrich M, Bokemeyer

C, Negaard H, Fankhauser C, Shamash J, Vaughn DJ, Sternberg CN, Heidenreich A,

Beyer J; International Germ Cell Cancer Classification Update Consortium.

Predicting Outcomes in Men With Metastatic Nonseminomatous Germ Cell Tumors

(NSGCT): Results From the IGCCCG Update Consortium. J Clin Oncol. 2021 May

10;39(14):1563-1574. doi: 10.1200/JCO.20.03296. Epub 2021 Apr 6. PMID: 33822655;

PMCID: PMC8099402.

162: Jake-Schoffman DE, McVay MA. Using the Design Sprint process to enhance and

accelerate behavioral medicine progress: a case study and guidance. Transl Behav

Med. 2021 May 25;11(5):1099-1106. doi: 10.1093/tbm/ibaa100. PMID: 33057685.

163: Impouma B, Mlanda T, Bukhari A, Sie Williams G, Farham B, Wolfe C, Mboussou

F, Botero Mesa S, Ngom R, Lee T, Keiser O. Information management practices in

the WHO African Region to support response to the COVID-19 pandemic. Epidemiol

Infect. 2021 May 26;149:e260. doi: 10.1017/S0950268821001242. PMID: 34036928;

PMCID: PMC8712935.

164: Lin PF, Naveed H, Eleftheriadou M, Purbrick R, Zarei Ghanavati M, Liu C.

Cataract service redesign in the post-COVID-19 era. Br J Ophthalmol. 2021

Jun;105(6):745-750. doi: 10.1136/bjophthalmol-2020-316917. Epub 2020 Jul 23.

PMID: 32703783.

165: Cheung CR, Finnemore A, Handforth J, Bohmer R, Christiansen N, Miller O;

Evelina London Children’s Hospital PIMS-TS Clinical and Study Group. Developing

new models of care at speed: learning from healthcare redesign for children with

COVID-related multisystem inflammation. Arch Dis Child. 2021 Jun;106(6):528-532.

doi: 10.1136/archdischild-2020-320358. Epub 2020 Oct 28. PMID: 33115714.

166: Taylor YJ, Kowalkowski M, Spencer MD, Evans SM, Hall MN, Rissmiller S,

Shrestha R, McWilliams A. Realizing a learning health system through process,

rigor and culture change. Healthc (Amst). 2021 Jun;8 Suppl 1:100478. doi:

10.1016/j.hjdsi.2020.100478. Epub 2021 Jun 23. PMID: 34175095.

167: Anton P. L'agilité organisationnelle en contexte de crise [Organisational

agility in a context of crisis]. Soins. 2021 Jun;66(856):61-65. French. doi:

10.1016/S0038-0814(21)00168-7. PMID: 34187659.

168: Steidtmann D, McBride S, Mishkind MC. Experiences of Mental Health

Clinicians and Staff in Rapidly Converting to Full-Time Telemental Health and

Work from Home During the COVID-19 Pandemic. Telemed J E Health. 2021

Jul;27(7):785-791. doi: 10.1089/tmj.2020.0305. Epub 2020 Dec 9. PMID: 33301354.

169: Panteli D, Maier CB. Regulating the health workforce in Europe:

implications of the COVID-19 pandemic. Hum Resour Health. 2021 Jul 10;19(1):80.

doi: 10.1186/s12960-021-00624-w. PMID: 34246288; PMCID: PMC8271310.

170: Grandone E, Tiscia GL, Mastroianno M, Larciprete G, Kovac M, Tamborini

Permunian E, Lojacono A, Barcellona D, Bitsadze V, Khizroeva J, Makatsarya A,

Cacciola R, Martinelli I, Bucherini E, De Stefano V, Lodigiani C, Colaizzo D, De

Laurenzo A, Piazza G, Margaglione M. Findings from a multicentre, observational

study on reproductive outcomes in women with unexplained recurrent pregnancy

loss: the OTTILIA registry. Hum Reprod. 2021 Jul 19;36(8):2083-2090. doi:

10.1093/humrep/deab153. PMID: 34195794.

171: Hammersley J, Mather C, Francis K. Lessons for Workforce Disaster Planning

from the First Nosocomial Outbreak of COVID-19 in Rural Tasmania, Australia: A

Case Study. Int J Environ Res Public Health. 2021 Jul 28;18(15):7982. doi:

10.3390/ijerph18157982. PMID: 34360276; PMCID: PMC8345614.

172: Herrmann S, Power B, Rashidi A, Cypher M, Mastaglia F, Grace A, McKinnon E,

Sarrot P, Michau C, Skinner M, Desai R, Duracinsky M. Supporting Patient-

Clinician Interaction in Chronic HIV Care: Design and Development of a Patient-

Reported Outcomes Software Application. J Med Internet Res. 2021 Jul

30;23(7):e27861. doi: 10.2196/27861. PMID: 34328442; PMCID: PMC8367117.

173: Bouza E, Cantón Moreno R, De Lucas Ramos P, García-Botella A, García-Lledó

A, Gómez-Pavón J, González Del Castillo J, Hernández-Sampelayo T, Martín-Delgado

MC, Martín Sánchez FJ, Martínez-Sellés M, Molero García JM, Moreno Guillén S,

Rodríguez-Artalejo FJ, Ruiz-Galiana J, De Pablo Brühlmann S, Porta Etessam J,

Santos Sebastián M. Síndrome post-COVID: Un documento de reflexión y opinión

[Post-COVID syndrome: A reflection and opinion paper]. Rev Esp Quimioter. 2021

Aug;34(4):269-279. Spanish. doi: 10.37201/req/023.2021. Epub 2021 Apr 20. PMID:

33878844; PMCID: PMC8329562.

174: Behne A, Krüger N, Beinke JH, Teuteberg F. Learnings from the design and

acceptance of the German COVID-19 tracing app for IS-driven crisis management: a

design science research. BMC Med Inform Decis Mak. 2021 Aug 9;21(1):238. doi:

10.1186/s12911-021-01579-7. PMID: 34372840; PMCID: PMC8350273.

175: Eren Vural I, Herder M, Graham JE. From sandbox to pandemic: Agile reform

of Canadian drug regulation. Health Policy. 2021 Sep;125(9):1115-1120. doi:

10.1016/j.healthpol.2021.04.018. Epub 2021 May 15. PMID: 34090723; PMCID:

PMC8123380.

176: Azizoddin DR, Adam R, Kessler D, Wright AA, Kematick B, Sullivan C, Zhang

H, Hassett MJ, Cooley ME, Ehrlich O, Enzinger AC. Leveraging mobile health

technology and research methodology to optimize patient education and self-

management support for advanced cancer pain. Support Care Cancer. 2021

Oct;29(10):5741-5751. doi: 10.1007/s00520-021-06146-4. Epub 2021 Mar 18. PMID:

33738594; PMCID: PMC8410657.

177: Sklar D, Yilmaz Y, Chan TM. What the COVID-19 Pandemic Can Teach Health

Professionals About Continuing Professional Development. Acad Med. 2021 Oct

1;96(10):1379-1382. doi: 10.1097/ACM.0000000000004245. PMID: 34292194; PMCID:

PMC8475641.

178: Soegaard Ballester JM, Bass GD, Urbani R, Fala G, Patel R, Leri D,

Steinkamp JM, Denson JL, Rosin R, Adusumalli S, Hanson CW, Koppel R, Airan-Javia

S. A Mobile, Electronic Health Record-Connected Application for Managing Team

Workflows in Inpatient Care. Appl Clin Inform. 2021 Oct;12(5):1120-1134. doi:

10.1055/s-0041-1740256. Epub 2021 Dec 22. PMID: 34937103; PMCID: PMC8695057.

179: Talasaz AH, Sadeghipour P, Aghakouchakzadeh M, Dreyfus I, Kakavand H,

Ariannejad H, Gupta A, Madhavan MV, Van Tassell BW, Jimenez D, Monreal M,

Vaduganathan M, Fanikos J, Dixon DL, Piazza G, Parikh SA, Bhatt DL, Lip GYH,

Stone GW, Krumholz HM, Libby P, Goldhaber SZ, Bikdeli B. Investigating Lipid-

Modulating Agents for Prevention or Treatment of COVID-19: JACC State-of-the-Art

Review. J Am Coll Cardiol. 2021 Oct 19;78(16):1635-1654. doi:

10.1016/j.jacc.2021.08.021. PMID: 34649702; PMCID: PMC8504484.

180: Orofino Vega P. Cuando la pandemia llama a la puerta de la salud laboral

[The pandemic knocks on the occupational health´s door.]. Rev Esp Salud Publica.

2021 Oct 22;95:e202110122. Spanish. PMID: 34675176.

181: Barr PJ, Haslett W, Dannenberg MD, Oh L, Elwyn G, Hassanpour S, Bonasia KL,

Finora JC, Schoonmaker JA, Onsando WM, Ryan J, Bruce ML, Das AK, Arend R, Piper

S, Ganoe CH. An Audio Personal Health Library of Clinic Visit Recordings for

Patients and Their Caregivers (HealthPAL): User-Centered Design Approach. J Med

Internet Res. 2021 Oct 22;23(10):e25512. doi: 10.2196/25512. PMID: 34677131;

PMCID: PMC8727051.

182: Abedian S, Kolivand P, Lornejad HR. Toward an Agile System: Iranian

Information System for Covid-19-Affected Patients Data Collection from Iranian

Hospitals. Stud Health Technol Inform. 2021 Oct 27;285:173-178. doi:

10.3233/SHTI210593. PMID: 34734870.

183: Posever N, Sehdev M, Sylla M, Mashar R, Mashar M, Abioye A. Addressing

Equity in Global Medical Education During the COVID-19 Pandemic: The Global

Medical Education Collaborative. Acad Med. 2021 Nov 1;96(11):1574-1579. doi:

10.1097/ACM.0000000000004230. PMID: 34261867; PMCID: PMC8541891.

184: Mao Z, Zou Q, Yao H, Wu J. The application framework of big data technology

in the COVID-19 epidemic emergency management in local government-a case study

of Hainan Province, China. BMC Public Health. 2021 Nov 4;21(1):2001. doi:

10.1186/s12889-021-12065-0. PMID: 34736445; PMCID: PMC8567122.

185: Lavie G, Weinstein O, Segal Y, Davidson E. Adapting to change: Clalit's

response to the COVID-19 pandemic. Isr J Health Policy Res. 2021 Nov

30;10(1):68. doi: 10.1186/s13584-021-00498-2. PMID: 34847927; PMCID: PMC8630513.

186: Goldin M, Giannis D, Diab W, Wang J, Khanijo S, Sharifova G, Cohen M, Lund

JM, Mignatti A, Gianos E, Tafur A, Lewis PA, Cohoon K, Kittelson JM, Lesser ML,

Sison CP, Rahman H, Ochani K, Hiatt WR, Dale RA, Anderson VE, Bonaca M, Halperin

JL, Weitz JI, Spyropoulos AC. Treatment-Dose LMWH versus

Prophylactic/Intermediate Dose Heparins in High-Risk COVID-19 Inpatients:

Rationale and Design of the HEP-COVID Trial. Thromb Haemost. 2021

Dec;121(12):1684-1695. doi: 10.1055/a-1475-2351. Epub 2021 Apr 6. PMID:

33823560.

187: Leppla L, Schmid A, Valenta S, Mielke J, Beckmann S, Ribaut J, Teynor A,

Dobbels F, Duerinckx N, Zeiser R, Engelhardt M, Gerull S, De Geest S; SMILe

study team. Development of an integrated model of care for allogeneic stem cell

transplantation facilitated by eHealth-the SMILe study. Support Care Cancer.

2021 Dec;29(12):8045-8057. doi: 10.1007/s00520-021-06328-0. Epub 2021 Jul 5.

PMID: 34224016; PMCID: PMC8550349.

188: Jennings J, Wundersitz DW, Sullivan CJ, Cousins SD, Tehan G, Kingsley MI.

Physical testing characteristics better explain draft outcome than in-game

movement profile in junior elite Australian rules football players. J Sci Med

Sport. 2021 Dec;24(12):1284-1289. doi: 10.1016/j.jsams.2021.07.005. Epub 2021

Jul 16. PMID: 34364809.

189: Talasaz AH, Sadeghipour P, Aghakouchakzadeh M, Kakavand H, Ariannejad H,

Connors JM, Hunt BJ, Berger JS, Van Tassell BW, Middeldorp S, Piazza G, Weitz

JI, Cushman M, Lip GYH, Goldhaber SZ, Bikdeli B. Use of novel antithrombotic

agents for COVID-19: Systematic summary of ongoing randomized controlled trials.

J Thromb Haemost. 2021 Dec;19(12):3080-3089. doi: 10.1111/jth.15533. Epub 2021

Sep 30. PMID: 34538017; PMCID: PMC8646701.

190: Malterud K, Kamps H. General practice - a fertile lagoon in the ocean of

medical knowledge. Scand J Prim Health Care. 2021 Dec;39(4):515-518. doi:

10.1080/02813432.2021.2004831. Epub 2021 Nov 16. PMID: 34783285; PMCID:

PMC8725917.

191: Sutton B, Clark TA. When the World Changes, Opportunities for Growth Come

Forward. Front Health Serv Manage. 2021 Dec 1;38(2):5-13. doi:

10.1097/HAP.0000000000000124. PMID: 34813511.

192: Hartney E, Melis E, Taylor D, Dickson G, Tholl B, Grimes K, Chan MK, Van

Aerde J, Horsley T. Leading through the first wave of COVID: a Canadian action

research study. Leadersh Health Serv (Bradf Engl). 2021 Dec 15;ahead-of-

print(ahead-of-print). doi: 10.1108/LHS-05-2021-0042. PMID: 34898142.

193: Torrente G, de Souza TQ, Tonaki L, Cardoso AP, Manickchand Junior L, da

Silva GO. Scrum Framework and Health Solutions: Management and Results. Stud

Health Technol Inform. 2021 Dec 15;284:290-294. doi: 10.3233/SHTI210725. PMID:

34920528.

194: Schneider BJ, Naidoo J, Santomasso BD, Lacchetti C, Adkins S, Anadkat M,

Atkins MB, Brassil KJ, Caterino JM, Chau I, Davies MJ, Ernstoff MS, Fecher L,

Ghosh M, Jaiyesimi I, Mammen JS, Naing A, Nastoupil LJ, Phillips T, Porter LD,

Reichner CA, Seigel C, Song JM, Spira A, Suarez-Almazor M, Swami U, Thompson JA,

Vikas P, Wang Y, Weber JS, Funchain P, Bollin K. Management of Immune-Related

Adverse Events in Patients Treated With Immune Checkpoint Inhibitor Therapy:

ASCO Guideline Update. J Clin Oncol. 2021 Dec 20;39(36):4073-4126. doi:

10.1200/JCO.21.01440. Epub 2021 Nov 1. Erratum in: J Clin Oncol. 2022 Jan

20;40(3):315. PMID: 34724392.

195: Romero L, Carneiro PB, Riley C, Clark H, Uy R, Park M, Mawokomatanda T,

Bombard JM, Hinckley A, Skapik J. Building capacity of community health centers

to overcome data challenges with the development of an agile COVID-19 public

health registry: a multistate quality improvement effort. J Am Med Inform Assoc.

2021 Dec 28;29(1):80-88. doi: 10.1093/jamia/ocab233. PMID: 34648005; PMCID:

PMC8524633.

196: Silva G, Bourne T, Hall G, Patel S, Rauf MQ, Vogel A, Carruthers A, Xu G.

Codeveloping an effective EMPA to maturity in an acute NHS Trust: an implementer

report. BMJ Health Care Inform. 2022 Jan;29(1):e100477. doi:

10.1136/bmjhci-2021-100477. PMID: 34983793; PMCID: PMC8728440.

197: INSPIRATION-S Investigators. Atorvastatin versus placebo in patients with

covid-19 in intensive care: randomized controlled trial. BMJ. 2022 Jan

7;376:e068407. doi: 10.1136/bmj-2021-068407. PMID: 34996756.

198: Fox KAA, Virdone S, Bassand JP, Camm AJ, Goto S, Goldhaber SZ, Haas S,

Kayani G, Koretsune Y, Misselwitz F, Oh S, Piccini JP, Parkhomenko A, Sawhney

JPS, Stepinska J, Turpie AGG, Verheugt FWA, Kakkar AK; GARFIELD-AF

investigators*. Do baseline characteristics and treatments account for

geographical disparities in the outcomes of patients with newly diagnosed atrial

fibrillation? The prospective GARFIELD-AF registry. BMJ Open. 2022 Jan

7;12(1):e049933. doi: 10.1136/bmjopen-2021-049933. PMID: 34996784; PMCID:

PMC8744109.

199: Melder A, Robinson T, Mcloughlin I, Iedema R, Teede H. Integrating the

complexity of healthcare improvement with implementation science: a longitudinal

qualitative case study. BMC Health Serv Res. 2022 Feb 19;22(1):234. doi:

10.1186/s12913-022-07505-5. PMID: 35183164; PMCID: PMC8858551.

200: Roche E, Lim C, Sayma M, Navaratnam A, Davis PJ, Ramnarayan P, Fraser J,

Kenny S; Paediatric Critical Care Society (PCCS), NHS England & NHS Improvement.

Learning lessons from the paediatric critical care response to the SARS-CoV-2

pandemic in England and Wales: a qualitative study. Arch Dis Child. 2022

Mar;107(3):e6. doi: 10.1136/archdischild-2020-320662. Epub 2021 Aug 20. PMID:

34417188; PMCID: PMC8384492.

201: Alibrahim A, Abdulsalam Y, Al Mutawa S, Behbehani H, Alhuwail D, Al Jenaei

S. Towards value-based healthcare: Establishing baseline pharmacy care costs for

diabetes management. Int J Health Plann Manage. 2022 Mar;37(2):790-803. doi:

10.1002/hpm.3370. Epub 2021 Oct 28. PMID: 34713500.

202: Årsand E, Muzny M, Bradway M, Muzik J, Hartvigsen G. Performance of the

first combined smartwatch and smartphone diabetes diary application study. J

Diabetes Sci Technol. 2015 May;9(3):556-63. doi: 10.1177/1932296814567708. Epub

2015 Jan 14. PMID: 25591859; PMCID: PMC4604524.

203: Buckingham CD, Adams A, Vail L, Kumar A, Ahmed A, Whelan A, Karasouli E.

Integrating service user and practitioner expertise within a web-based system

for collaborative mental-health risk and safety management. Patient Educ Couns.

2015 Oct;98(10):1189-96. doi: 10.1016/j.pec.2015.08.018. Epub 2015 Aug 14. PMID:

26320825.

204: Ahern DK, Parker D, Eaton C, Rafferty C, Wroblewski J, Goldman R. Patient-

facing Technology for Identification of COPD in Primary Care. J Innov Health

Inform. 2016 Jul 15;23(2):824. doi: 10.14236/jhi.v23i2.824. PMID: 27869583.

205: Hekler EB, Klasnja P, Chevance G, Golaszewski NM, Lewis D, Sim I. Why we

need a small data paradigm. BMC Med. 2019 Jul 17;17(1):133. doi:

10.1186/s12916-019-1366-x. PMID: 31311528; PMCID: PMC6636023.

206: Kohavi R, Tang D, Xu Y, Hemkens LG, Ioannidis JPA. Online randomized

controlled experiments at scale: lessons and extensions to medicine. Trials.

2020 Feb 7;21(1):150. doi: 10.1186/s13063-020-4084-y. PMID: 32033614; PMCID:

PMC7007661.

207: Donato M, Carini FC, Meschini MJ, Saubidet IL, Goldberg A, Sarubio MG,

Olmos D, Reina R. Consensus for the management of analgesia, sedation and

delirium in adults with COVID-19-associated acute respiratory distress syndrome.

Rev Bras Ter Intensiva. 2021 Jan-Mar;33(1):48-67. doi:

10.5935/0103-507X.20210005. PMID: 33886853; PMCID: PMC8075332.

208: Fries JA, Steinberg E, Khattar S, Fleming SL, Posada J, Callahan A, Shah

NH. Ontology-driven weak supervision for clinical entity classification in

electronic health records. Nat Commun. 2021 Apr 1;12(1):2017. doi:

10.1038/s41467-021-22328-4. PMID: 33795682; PMCID: PMC8016863.

209: Burner ER, Menchine MD, Kubicek K, Robles M, Arora S. Perceptions of

successful cues to action and opportunities to augment behavioral triggers in

diabetes self-management: qualitative analysis of a mobile intervention for low-

income Latinos with diabetes. J Med Internet Res. 2014 Jan 29;16(1):e25. doi:

10.2196/jmir.2881. PMID: 24476784; PMCID: PMC3936269.

210: van Mierlo T, Fournier R, Jean-Charles A, Hovington J, Ethier I, Selby P.

I'll txt U if I have a problem: how the Société Canadienne du cancer in Quebec

applied behavior-change theory, data mining and agile software development to

help young adults quit smoking. PLoS One. 2014 Mar 19;9(3):e91832. doi:

10.1371/journal.pone.0091832. PMID: 24647098; PMCID: PMC3960136.
